# The Minimum Effective Training Dose Required for 1RM Strength in Powerlifters

**DOI:** 10.3389/fspor.2021.713655

**Published:** 2021-08-30

**Authors:** Patroklos Androulakis-Korakakis, Nick Michalopoulos, James P. Fisher, Justin Keogh, Jeremy P. Loenneke, Eric Helms, Milo Wolf, Greg Nuckols, James Steele

**Affiliations:** ^1^Faculty of Sport, Health, and Social Sciences, Solent University, Southampton, United Kingdom; ^2^Department of Physics, University of Patras, Patras, Greece; ^3^Faculty of Health Sciences and Medicine, Bond University, Gold Coast, QLD, Australia; ^4^Cluster for Health Improvement, Faculty of Science, Health, Education and Engineering, University of the Sunshine Coast, Maroochydore, QLD, Australia; ^5^Kasturba Medical College, Mangalore, India; ^6^Manipal Academy of Higher Education, Manipal, India; ^7^Sports Performance Research Institute New Zealand (SPRINZ), Auckland University of Technology, Auckland, New Zealand; ^8^Kevser Ermin Applied Physiology Laboratory, Department of Health, Exercise Science, and Recreation Management, The University of Mississippi, Oxford, MS, United States; ^9^Stronger by Science LLC, Chapel Hill, NC, United States

**Keywords:** powerlifting, strength, minimum dose, squat, bench press, deadlift

## Abstract

The aim of this multi-experiment paper was to explore the concept of the minimum effective training dose (METD) required to increase 1-repetition-maximum (1RM) strength in powerlifting (PL) athletes. The METD refers to the least amount of training required to elicit meaningful increases in 1RM strength. A series of five studies utilising mixed methods, were conducted using PL athletes & coaches of all levels in an attempt to better understand the METD for 1RM strength. The studies of this multi-experiment paper are: an interview study with elite PL athletes and highly experienced PL coaches (*n* = 28), an interview and survey study with PL coaches and PL athletes of all levels (*n* = 137), two training intervention studies with intermediate-advanced PL athletes (*n* = 25) and a survey study with competitive PL athletes of different levels (*n* = 57). PL athletes looking to train with a METD approach can do so by performing ~3–6 working sets of 1–5 repetitions each week, with these sets spread across 1–3 sessions per week per powerlift, using loads above 80% 1RM at a Rate of Perceived Exertion (RPE) of 7.5–9.5 for 6–12 weeks and expect to gain strength. PL athletes who wish to further minimize their time spent training can perform autoregulated single repetition sets at an RPE of 9–9.5 though they should expect that strength gains will be less likely to be meaningful. However, the addition of 2–3 back-off sets at ~80% of the single repetitions load, may produce greater gains over 6 weeks while following a 2-3-1 squat-bench press-deadlift weekly training frequency. When utilizing accessory exercises in the context of METD, PL athletes typically utilize 1–3 accessory exercises per powerlift, at an RPE in the range of 7–9 and utilize a repetition range of ~6–10 repetitions.

## Introduction

Increased muscular strength is associated with a multitude of potential benefits including improved physical performance, decreased morbidity/mortality risk, and possible increases in sports performance (Westcott, 2012; Suchomel et al., 2016). Powerlifting (PL) is a strength sport in which maximal strength determines competitive success. PL is based on 3 barbell lifts (the “powerlifts”): the squat (SQ), bench press (BP), and deadlift (DL) (Androulakis-Korakakis et al., 2018). In competition, a PL athlete is allowed three 1-repetition-maximum (1RM) attempts at each of the powerlifts, with the goal of achieving the highest possible PL total (i.e., the sum of their highest successful lifts). In competitive PL, one of the most common competitive formats is “raw” where SQ, BP, and DL compressive suits, shirts or knee wraps are not permitted, only allowing use of knee sleeves, wrist wraps, and a belt.

The powerlifts are common resistance training exercises, not only used within PL, but also other strength sports (e.g.,: Strongman), and in training by athletes of non-strength related sports and recreational lifters (Jones et al., 2016; Vecchio et al., 2018). The powerlifts are multi-joint exercises utilizing multiple major muscle groups and thus are considered an efficient modality of resistance training (Gentil et al., 2016; Paoli et al., 2017).

When planning their training, PL athletes manipulate training variables (e.g., sets, repetitions, and load) and often utilize different periodization or programming schemes to do so (Zourdos et al., 2016). PL athletes often manipulate training volume depending on their desired physiological outcomes, as well as how close they are to a competition (Pritchard et al., 2016). For example, a recent review by Travis et al. (2020) suggested that PL athletes tapering for competition should reduce training volume by ~30–70% while training with heavy loads (>85% 1RM). Some evidence supports a greater training volume producing larger increases in muscle hypertrophy (Schoenfeld et al., 2019) but the relationship between training volume and maximal strength is unclear and warrants further research (Ralston et al., 2017, 2018; Lopez et al., 2020). Additionally, the relationship between muscle hypertrophy and strength has also been questioned within the literature (Loenneke et al., 2019).

A concept that has been explored in recent years is the minimum effective training dose (METD) for 1RM strength (Androulakis-Korakakis et al., 2019). METD is essentially the lowest training stimulus an individual can be exposed to and still make meaningful strength increases. A systematic review by Androulakis-Korakakis et al. (2019) examined the current literature around the concept of METD, specifically focusing on studies that utilized the powerlifts and found that performing a single set of 6–12 repetitions with loads ranging from 70 to 85% 1RM, 2–3 times per week with a high intensity of effort (reaching volitional or momentary failure) for 8–12 weeks can produce suboptimal, yet statistically significant increases in SQ and BP 1RM strength. The review highlighted that currently, there is no data regarding METD for the DL, or studies with women and highly strength trained athletes; but, it was noted that PL athletes could potentially benefit from the concept of METD. Further, a pilot study by Androulakis-Korakakis et al. (2018) found that competitive PL athletes were able to increase their peri-training 1RM strength using a very low training volume protocol consisting of a total of two heavy single repetitions per week for the SQ, three heavy repetitions per week for the BP and one heavy single repetition per week for the DL (excluding repetitions performed as part of the athletes' warm-up sets).

Attaining meaningful increases in maximal strength by utilizing the lowest training dose possible, especially in the context of multi-joint exercises like the powerlifts, may not only benefit PL and strength sport athletes, but also athletes of other sports and recreationally active individuals. Understanding the overall utility of the concept of METD for 1RM strength in powerlifters along with its limitations and considerations for application may allow PL athletes to increase maximal strength by doing less training volume, allowing for more training flexibility without impairments in PL performance. A METD approach may be particularly useful for PL athletes who due to work or family commitments have a limited time to allocate to training. Additionally, it may allow PL athletes to periodically reduce training stress, alleviate burnout while potentially minimizing injury risk. Therefore, in this paper we describe the results of a series of studies utilizing mixed methods aimed at exploring the concept of the METD in powerlifters.

## Overview of Studies

To address the research question in hand, we conducted a total of five studies. A summary of each study can be found in Table 1.

**Table 1:** Summary of studies.

**Study**	***n***	**Study type**	**Sample**	**Purpose**
1	28	Semi-structured interviews	Elite PL athletes and experienced PL coaches	To understand the concept of METD, its utility, applicability, and limitations
2	137 and 28*	Survey and semi-structured interviews	PL athletes and coaches of all levels, elite PL athletes, experienced PL coaches	To understand what PL athletes and coaches regard as meaningful strength increases over a training period of 6 weeks to assist with the data analysis and interpretation of the training intervention studies
3	16	Training Intervention	Intermediate-advanced PL athletes	To explore the effect of two different “METD” training protocols on 1RM strength in PL athletes over 6 weeks.
4	9	Training Intervention	Beginner-intermediate PL athletes	To explore the effect of two different “METD” training protocols on 1RM strength in PL athletes over 6 weeks.
5	58	Survey	Intermediate-advanced competitive PL athletes	To understand the “METD” practices of competitive PL athletes as well as the reasons for not training with a “METD” approach

* *Denotes participants from study 1*.

### Study 1—The Minimum Effective Training Dose for 1RM Strength in Powerlifters: Semi-structured Interviews With Elite PL Athletes and Highly Experienced PL Coaches

#### Materials and Methods

##### Design and Approach to the Problem

Semi-structured interviews with highly experienced PL coaches and elite PL athletes were conducted to explore how these populations understand the concept of the METD for 1RM strength in powerlifters. Semi-structured interviews allowed for the concept of a METD to be explored inductively from a multitude of perspectives, addressing some of the potential limitations of the training intervention studies described later in the manuscript. Semi-structured interviews have previously been employed with elite PL athletes and coaches and can help encapsulate the richness of their experiences and practices, allowing others to learn from them (Pritchard et al., 2016).

The semi-structured interviews were designed using a set of guiding questions but allowed for some flexibility for participants to expand on their answers and further discuss their experience and understanding of the METD. The questions were designed around the research questions of the study—understanding METD as a concept, its practical use, the length of its effectiveness, appropriate use timing, and other considerations around its overall utility and applicability.

Prior to commencing the interviews, the study was approved by the Solent University Health, Exercise, and Sport Science Ethics Committee (reference: *andrp2020*).

##### Participants

To ensure that the PL coaches recruited for the study were of sufficient experience, they had to be coaching competitive raw PL athletes for a minimum of 3 years who were competing at the national level or higher in a federation affiliated with the International Powerlifting Federation (IPF). There were no inclusion criteria specified for biological sex for the PL coaches, but all PL coaches were required to have experience working with both male and female PL athletes. To ensure that the PL athletes recruited for the study were high level athletes they had to meet the following inclusion criteria: have a Wilks score (a formula commonly used to determine strength relative to body mass in powerlifting) of at least 450, compete raw at the national level in an IPF-affiliated federation or raw at the international level at a non-IPF affiliated federation. The Wilks score, calculated through the Wilks formula, multiplies a PL athlete's lift by an index based on body mass, allowing for the comparison of different-mass PL athletes on the same powerlifts (Vanderburgh and Batterham, 1999). Despite the Wilks score being replaced by a newer formula, IPF points at IPF competitions, there is recent evidence to suggest that the Wilks formula is more efficient at comparing men's weight classes and that the IPF's decision to replace it for IPF points could not be validated (Ferland et al., 2020). In contrast to the PL coaches, we aimed for an approximately equal number of female and male PL athletes. Sample size was convenience-based and justified based on feasibility expectations given the authors' access to the population to be sampled (i.e., a resource constraints based justification; Lakens, 2021). Further, we considered previous research with highly experienced coaches and athletes using samples ranging from 5 to 11 participants per population (Leidl et al., 2009; Kim et al., 2016; Pritchard et al., 2016) as a heuristic guide.

Following recruitment *via* email through personal networks and social media, 23 potential PL coaches were identified as participants. From the 23 PL coaches contacted, three participants did not respond to the study invitation and two participants were excluded due to not having sufficient experience which resulted in 18 PL coaches participating in the study. Sixteen potential PL athletes were contacted through similar approaches. Of the 16 PL athletes contacted, six did not respond to the study invitation and 10 raw PL athletes participated. Despite not being set as part of the inclusion criteria, nine out of the 10 PL athletes competed at drug-tested federations (IPF-affiliated federations). Prior to involvement in the study, the aims, details, and potential risks of participating in the study were presented to participants and informed consent was obtained. The PL coaches' characteristics including age, total athletes coached, powerlifting coaching experience, and IPF world and IPF national championship first place finishers can be found in Table 2. The PL athletes' characteristics including age, competition weight, competition experience, best competition SQ, BP, DL, and PL Total can all be found on Table 3. In addition to the PL athletes' characteristics, their IPF national and world first place finishes along with their IPF records and all-time-world-records (ATWR) can be found on Table 3.

**Table 2:** PL coaches' characteristics.

**Characteristic**	
Age	30.3 ± 6.5
Total Athletes Coached* (Sum)	3620
Powerlifting Coaching Experience (years)	8.7 ± 5.3
RAW (%)	90
RAW and Equipped (%)	10
International (IPF) (%)	90
National (IPF) (%)	10
IPF World Championships 1st places* (Sum)	32
IPF European Championships 1st places* (Sum)	18
IPF National Championships 1st places* (Sum)	244
National teams coached* (Sum)	11

*Results are mean ± SD*.

* *Approximate numbers based on the estimation of the PL coaches*.

**Table 3:** PL athletes' characteristics.

**Characteristic**	**Male**	**Female**
Age	30 ± 10.4	37.4 ± 11.6
Weight (kg)	96.4 ± 13.8	57.4 ± 5.5
Comp Experience (years)	5.8 ± 3.5	12.8 ± 11.8
Best Comp 1RM SQ (kg)	266 ± 9.6	175.6 ± 11.8
Best Comp 1RM BP (kg)	173.6 ± 12.2	101.5 ± 27
Best Comp 1RM DL (kg)	312.8 ± 29.2	195.7 ± 29.2
Best Comp Total (kg)	752.4 ± 38.7	462.5 ± 58.3
IPF World Championships (Sum)	1	13
IPF National Championships (Sum)	9	39
IPF National SQ Record (Sum)	0	1
IPF National BP Record (Sum)	0	2
IPF National DL Record (Sum)	3	0
IPF National Total Record (Sum)	1	0
IPF World SQ Record (Sum)	0	1
IPF World BP Record (Sum)	0	1
IPF World Total Record (Sum)	1	1
ATWR BP**	0	1
ATWR DL**	0	1
ATWR Total**	0	1

*Results are mean ± SD, Comp, Competition*.

** *Compared across all federations using data from the database openpowerlifting.org, IPF, International Powerlifting Federation; ATWR, All Time World Record*.

##### Procedures

After obtaining informed consent participants were contacted and interviewed using an online video conferencing platform (for convenience the specific platform used varied by participant based on their accessibility). Prior to any recording, informed consent was obtained for this specifically (participants were informed of whether the services used offered end to end encryption and data privacy). The interviews lasted 15–58 min and were recorded using the software OBS (https://obsproject.com/) for transcription purposes. Interview files were then converted to MP3 audio files using a video converter (Wondershare Technology Co, Shenzhen, China) and then uploaded to an online artificial intelligence transcription service, Otter (https://otter.ai) on the private account of the primary investigator. Following the automatic transcription of all interviews, a process that lasted ~20 min, the interview transcripts were downloaded as text files and permanently deleted from the transcription service. The generated transcriptions were checked for accuracy and corrected by two of the investigators using the original audio file, where required.

Participants were asked demographic and coaching/performance questions before being asked open-ended questions around the concept of the METD required to increase 1RM strength in PL athletes. Participants were also asked about what they would consider a meaningful change in strength over a 6 weeks training period, the results of which are described in study 2 of this manuscript. The questions asked during the semi-structured interviews can be found in the Supplementary Material.

##### Analyses

Qualitative data was analyzed using a similar thematic content analysis to Pritchard et al. (2016). The thematic content analysis of the transcripts was conducted using the NVivo 12 software package (QSR International, Cambridge, MA, USA). The participants' interview transcripts were organized into broad themes to assist in collating together all the obtained responses. A label was assigned to each broad theme to identify its content (e.g.,: The minimum effective dose for 1RM strength—in practice). The number of broad themes was determined by the content of the participants' interviews and what was discussed. Following the identification of broad themes, the coding process began, identifying individual text units in each participants' interview responses. The text units were compared with other text units under the identified broad themes which enabled subthemes to emerge. Themes and subthemes were classified similarly to Hill et al. (2005). The classifications used were the following: general, themes applying to all or all but one participant; typical, themes applying to more than half the participants, but less than general; variant, themes applying to two or more participants, but less than typical (Pritchard et al., 2016).

##### Validity and Reliability

Two types of triangulation were used to establish validity and reliability. Firstly, PL coaches and PL athletes of different sexes across different weight classes, of different competitive experiences, and of different strength levels were recruited showing external validity as similar themes and subthemes emerged amongst these individuals. As a second form of triangulation, two other researchers were asked to evaluate the identified themes and subthemes to ensure that the themes and subthemes were indicative of the data collected.

#### Results

The broad themes and subthemes as well as their respective sample representativeness can be found below in Table 4. The participants' views and percentage of representativeness for each theme are further analyzed below. The participants' views are presented in italicized quotes.

**Table 4:** METD interview themes/subthemes coaches and athletes.

**Theme**	**Sample representativeness** **(% of sample and number of participants)**	
	**Athletes (*n* = 10)**	**Coaches (*n* = 18)**
**METD for 1RM strength as a concept**		
**Subtheme(s)**		
Interesting/Useful concept	30% (*n* = 3)	27% (*n* = 5)
Important concept to ensure long-term success	10% (*n* = 1)	27% (*n* = 5)
**METD for 1RM strength—in practice**		
**Subtheme(s)**		
A few high load sets per week	80% (*n* = 8)	61% (*n* = 11)
1–5 repetitions with a heavy load per main set	70% (*n* = 7)	55% (*n* = 10)
The BP requires a higher training frequency	30% (*n* = 3)	22% (*n* = 4)
RPE 8+ for the main sets	10% (*n* = 1)	27% (*n* = 5)
SQ trained one time per week	30% (*n* = 3)	11% (*n* = 2)
SQ trained two times per week	0%	22% (*n* = 4)
BP trained one per week	0%	11% (*n* = 2)
BP trained 2–3 times per week	10% (*n* = 1)	22% (*n* = 4)
DL trained one time per week	20% (*n* = 2)	27% (*n* = 5)
**METD for 1RM strength—length**		
**Subtheme(s)**		
Effective for ~6–12 weeks	50% (*n* = 5)	50% (*n* = 9)
As long as it remains effective	0%	22% (*n* = 4)
**METD for 1RM strength—when**		
**Subtheme(s)**		
When time is limited	30% (*n* = 3)	27% (*n* = 5)
Pre-competition	30% (*n* = 3)	5% (*n* = 1)
When not feeling 100%	20% (*n* = 2)	11% (*n* = 2)
**METD for 1RM strength—considerations**		
**Subtheme(s)**		
Suboptimal—“why do less when you can do more”	20% (*n* = 2)	11% (*n* = 2)
Change of mindset from more is always better	30% (*n* = 3)	0%

The thematic analysis for subthemes with sample representativeness below 50% can be found under “Thematic analysis Study 1” in the Supplementary Material.

**Broad Theme:** METD for 1RM strength—in practice.

**Subtheme:***A few high load sets per week*.

##### PL Athletes

Eighty percent of PL athletes expressed that “*a few heavy sets per week”* per powerlift may be enough to make meaningful strength increases. Other example responses included variants on this when asked what METD would look like in practice. PL athletes responded that they would use “*2-3 heavy sets per week”* as well as “*2-3 hard sets on each powerlift”* would suffice. Additionally, they mentioned past experiences where they “*have seen great progress with just a few heavy sets per week.”*

##### PL Coaches

Though fewer than the PL athletes, more than half (61%) of PL coaches also expressed the view that a few high load sets per week could be enough to make meaningful strength increases. PL coaches expanded on how they “*would just go straight for low rep high intensity work, so we might do triples, doubles or singles at RPE 8 and up maybe twice a week, or something like that. And so you could probably get away with increasing someone's strength off of six reps a week, if you really need to”* with some coaches suggesting as low as “*two or three heavy singles will probably get the job done, for someone without much experience with high load training.”* Some coaches also touched on how they would vary the type of high load sets per week, utilizing heavy single repetitions and less heavy, but still high load, back-off sets of multiple repetitions. For example, one coach expanded on how they “*would probably have them work up to a relatively hard single at the beginning of each one of the powerlifts on separate days as far apart as they can. For example, Monday, Thursday, have them working to a relatively heavy single, like the range we mentioned before was RPE 9-9.5. And so that's probably about what I would be looking for.... And then I would have them do a small number of back-off sets. You know, something in the range of three sets of two or three sets of three, at a relatively high load”* while others mentioned that they “*could just, you know, work up to a single, double or triple and back off for a couple of sets and walk away.”*

**Subtheme:** *1–5 repetitions for the working sets*

##### PL Athletes

Seventy percent of PL athletes expressed that their working sets would be composed of ~1–5 repetitions. They mentioned that when other PL athletes try to implement a METD approach they “*should try to hit within the rep range of five”* as it was felt to be “*the perfect number as far as getting a little bit of volume and but also being able to hit max weights.”* Some also drew on past experiences describing what they have “*done in the past, and I know has worked in terms of my strength”* noting for example having “*had blocks where I've done [mainly] sort of doubles, or triples”* as well as remembering seeing “*great progress with just a few heavy sets on the squat and deadlift with mostly singles and triples.”* Though some individual variability was noted regarding the efficacy of this with one noting for example “*for the bench I sometimes have to push it a bit more like 2–3 times per week and do more repetitions around 5 but that could just be me.”* Other PL athletes mentioned how they would “*work up to either a top set, you know, a low rep set to set so either like, one, two or three reps, even as high as five and then accumulate some volume, do some drop sets, you know, maybe like three sets of something or four sets of something at a lower weight to accumulate volume”* as well as giving more general responses on how they would “*focus more on heavy loads, so let's say 90% 1RM, but then you know, not that many reps or sets.”*

##### PL Coaches

More than half of PL coaches expressed that they would prescribe working sets of ~1–5 repetitions when using a METD approach. They mentioned that they would “*go straight to for low rep high intensity work [sic], so we might do triples, doubles or singles”* with some other PL coaches expressing that they “*would want the reps to be only singles, if not like, maybe doubles.”* The concept of utilizing single repetitions and back-off sets was also mentioned by some coaches as they discussed how they would advise PL athletes to “*work up to a heavy single like RPE 8-9, and then 10–15% of that for like three to four reps to two to three sets three to four reps at like at the weight of the top single”* and that they “*would probably have them work up to a relatively hard single at the beginning of each session…. and then I would have them do a small number of back-off sets. You know, something in the range of three sets of two or three sets of three, at a relatively high intensity.”*

**Broad Theme:** METD—length of effectiveness.

**Subtheme**: *6–12 weeks*.

##### PL Athletes

Fifty percent of PL athletes expressed that they believed a METD approach would be effective for ~6–12 weeks. Some PL athletes were on the lower side of the range for example noting “*say maybe sort of 6–8 weeks maybe is what I'd usually sort of do in terms of how long I would structure kind of a lower volume phase”* and that they would expect such an approach to stop being effective “*at about 8 weeks.”* Other PL athletes expressed that they would expect such an approach to work for longer periods of time than 6 weeks expressing that “*12 weeks seems like a fair approximation.”*

##### PL Coaches

Similarly, to the PL athletes, 50% of PL coaches expressed that they believed a METD approach would be effective for ~6–12 weeks. Some coaches expressed that the length of effectiveness could be extended past 6 weeks if appropriate adjustments were made at the 6-week mark. They made statements like “*If you didn't change anything meaningful, so if you didn't adjust the reps or the load, I would say 6 weeks before something has to change, you have to reset and start a progression over. If you started at triples, and work your way down to singles, then you could extend that out to maybe 10–12 weeks.”* Some others expanded on how they could potentially “*lose buy-in from the lifter*” if they extended METD for more than 6 weeks. Other PL coaches were somewhere in the middle of the 6–12 weeks range expressing how they would implement such an approach for “*8 weeks, which is typically one to two mesocycles”* and that they “*would implement it for up to 8 weeks.”*

#### Discussion

The results of study 1 provide a clearer understanding around the utility, applicability, and limitations of the concept of METD required to increase 1RM strength in powerlifters. The most common subtheme that emerged under the theme “concept” was that METD is deemed interesting and useful. Indeed, 27% of PL athletes considered the METD an important concept when attempting to ensure long-term success. The importance of long-term success may be linked to the subtheme “when time is limited,” where 30% PL coaches and 27% PL athletes mentioned that they would utilize such a training approach if time was limited. The ability to make meaningful, albeit not optimal, strength increases when longer training sessions are not possible may allow PL athletes to continue making progress while also ensuring they are not doing more work than they can recover from. Though, at present, it is worth noting that there is only limited evidence on the role of overtraining in resistance training specifically (Grandou et al., 2020). Another potential use for METD is in competition preparation where time is limited with 30% of PL athletes expressing that they would consider this. The consensus from recent reviews on tapering and peaking maximal strength for powerlifting performance suggest that a reduction in volume, yet maintenance of training loads, may be optimal (Pritchard et al., 2015; Travis et al., 2020). Indeed, in a similar interview-based study, albeit focused on tapering specifically, Pritchard et al. (2016) noted that this was the approach typically employed by powerlifters and thus, corroborates our findings here relating to the METD concept more generally.

In regards to how a METD is practically employed, 80% of PL athletes and 61% of PL coaches expressed that the METD may consist of a few heavy load sets per week, with 70% of PL athletes and 55% of coaches expressing that 1–5 repetitions per main set may be enough to attain meaningful strength increases over the period of 6–12 weeks (expressed by 50% of PL athletes and PL coaches). Heavy loads may be more beneficial for maximal strength increases, especially when testing maximal strength *via* a 1RM test, as is required in the sport of PL (Fisher et al., 2020; Schoenfeld et al., 2021). The repetition range expressed by the PL athletes and coaches during the interviews may allow PL athletes to practice the powerlifts using heavy loads that will then translate to better strength gains when strength is assessed at a competition using a 1RM test. Indeed, a series of studies has examined training consisting of single 1RM lifts (referred to as “practicing the test”) compared to more traditional resistance training, finding similar improvements in 1RM strength (Dankel et al., 2017, 2020; Mattocks et al., 2017; Buckner et al., 2021). Some of the PL athletes expressed in interviews that they would solely train using single repetitions, which would in essence be practicing the test for a PL athlete. A case study by Zourdos et al. (2015) found that 2 PL athletes and an Olympic Weightlifting athlete were able to increase their SQ 1RM after performing daily 1RM training for 37 consecutive days by 12.5, 21, and 13.5 kg, respectively. The Zourdos et al. (2015) case study may not be completely indicative of METD training as it entailed daily sessions, included some follow up volume sets and it also did not include the other two powerlifts, but it demonstrates that merely “practicing the test” can produce meaningful strength gains even in strength athletes. A small pilot study by Androulakis-Korakakis et al. (2018) has also explored this approach in PL athletes preparing for competition. Five PL athletes were able to increase their PL total peri-training intervention (around the 5–7 weeks mark) utilizing only a few sets of single repetitions per week, but during competition (and after 10 weeks of training) three out of five participants actually saw a decrease to their PL total. However, they note that while the competition setting for post-intervention outcomes offered ecological validity, weight selection for competition attempts may have impacted final performance. Nevertheless, the study suggested that it is possible to produce short-term improvements with a METD approach in essence just “practicing the test,” but that after a certain point a PL athlete may require a greater training stimulus, perhaps including more volume or frequency to continue to make meaningful progress. Considering the results of this interview study, PL athletes' and coaches' conceptualization and application of a METD based approach was broadly reflective of the current evidence on the topic. In specific circumstances such as when busy, or during competition preparation, meaningful strength gains may be possible with a METD approach. However, it is not fully clear what is meant by PL athletes and coaches when considering “meaningful” changes. Thus, as suggested by Steele et al. (2020), in order to aid in the interpretation of intervention research on the METD, it is necessary to understand what is considered “meaningful” by these populations.

### Study 2—Meaningfulness of Strength Changes Following a 6-Week Training Protocol: A Survey and Interview Study

#### Materials and Methods

##### Design and Approach to the Problem

A survey of PL athletes of all levels (regional, national, and international) and semi-structured interviews with highly experienced PL coaches and elite PL athletes were conducted to better understand what is regarded as a meaningful increase in SQ, BP, DL, and PL total strength over 6 weeks. An expert elicitation of minimal important effect approach may allow for better interpretation of the intervention results (Steele et al., 2020), and so this was conducted primarily to inform interpretation of the results from the two training studies that are described in detail later in the manuscript. Prior to commencing the survey and interviews, the study was approved by the Solent University Health, Exercise, and Sport Science Ethics Committee (reference: *andrp2020*).

##### Participants

Participants for the survey part of this study were recruited through personal networks and social media with the aim of reaching as many raw PL athletes and PL coaches as possible. As such, the sample size justification was resource constraint based (Lakens, 2021) in that we were constrained by the number of participants willing to respond to the survey. The inclusion criteria for the interview participants can be found under the methods section of study 1 as the same participants were involved. Participants of the survey study were required to be a PL coach or raw PL athlete with no inclusion criteria set for strength level, competition experience, or federation. A total of 137 PL coaches and athletes completed the survey. Survey participant characteristics can be found in Table 5. Participant information for the interview part of this study can be found under Study 1 methods.

**Table 5:** Study 2 survey participant characteristics.

***n*** **= 137**	**Age (years)**	**% of participants**	**Training experience (years)**	**Coaching experience (years)**
Athlete	27.5 ± 8	66.9	5.6 ± 6	N/A
Athlete and Coach	31.8 ± 5.8	25.8	14 ± 6.7	9.5 ± 4.2
Coach	28.9 ± 8.3	7.3	7.9 ± 5.1	5.2 ± 5.9
**Participant Level** *Coaching, competing, or both*	**Regional**	**National**	**International**	
**% of participants**	69.5	47.4	18.5	

*Results are mean ± SD*.

##### Procedures

The procedures for the interview participants are described in the methods section of Study 1. The survey participants were informed at the beginning of the survey about the aims and potential risks of the study and were asked to provide informed consent prior to completing the survey. Following this they answered demographic and training/coaching experience questions before then answering in kg what they would consider to be a meaningful change in strength for the SQ, BP, DL, and PL total over a 6 weeks period of training. We deliberately did not define “meaningful” for the participants as we wanted it to be left open to their own idiosyncratic interpretation and the interview portion of this study offered additional insight into what people considered “meaningful.” Further, the justification for selecting 6 weeks as the training period was because the intervention studies conducted and described later were of this duration given the peri-training intervention results of the pilot study of “daily max” training by Androulakis-Korakakis et al. (2018). The survey questions can be found in the Supplementary Material.

##### Statistical Analyses

The analysis approach for the qualitative data obtained from the interviews can be found under the “Methods” section of Study 1. For the survey responses, descriptive statistics (means and standard deviations), and % of respondents were calculated.

#### Results

The descriptive results of the survey respondents for what they regarded as a meaningful strength increase for SQ, BP, DL, and PL total strength can be found in Table 6.

**Table 6:** Meaningful strength increases in 6 weeks.

***n*** **= 137**	**SQ 1RM (kg)**	**BP 1RM (kg)**	**DL 1RM (kg)**	**Total (kg)**
Athlete (*n* = 99)	6.8 ± 3.9	4.2 ± 3.1	8.2 ± 4.8	17.7 ± 12.1
Athlete and Coach (*n* = 31)	8.3 ± 6.3	5.1 ± 3.6	8.7 ± 6	16.8 ± 16.3
Coach (*n* = 7)	7.8 ± 7.6	4.6 ± 3.6	7.5 ± 5.1	16.6 ± 11.5
All	7.1 ± 5.1	4.4 ± 3.3	8.1 ± 5	17.5 ± 12.1

*Results are mean ± SD, “Athlete & Coach” denotes participants who were both a PL athlete and a PL coach*.

The broad themes and subthemes as well as their respective sample representativeness can be found in Table 7. The participants' views and percentage of representativeness for each theme are further analyzed below. The participants' views are presented in Italicized quotes.

**Table 7:** Study 2 interview themes and subthemes.

**Theme**	Sample representativeness* (% of sample and number of participants)	
	**Athletes (*n* = 10)**	**Coaches (*n* = 18)**
**Meaningfulness of strength changes in 6 weeks**		
Any measurable change in 6 weeks may be meaningful	50 (*n* = 5)	50 (*n* = 9)
A 5–10 kg increase on the SQ may be meaningful	30 (*n* = 3)	33 (*n* = 6)
A 2.5–5 kg increase on the BP may be meaningful	20 (*n* = 2)	33 (*n* = 6)
A 5–10 kg increase on the DL may be meaningful	30 (*n* = 3)	16 (*n* = 3)
A 2.5–5 kg increase per powerlift may be meaningful	10 (*n* = 1)	16 (*n* = 3)
A 2+% increase per powerlift may be meaningful	N/A	33% (*n* = 6)
**Factors affecting the magnitude of meaningfulness of strength changes in 6 weeks**		
Strength level and experience of the athlete	20 (*n* = 2)	72 (*n* = 13)
Time of the training season (competition preparation vs. off-season)	50 (*n* = 5)	N/A
Bodyweight of the athlete	N/A	33 (*n* = 6)

*The participants of the interviews are the PL athletes and coaches from study 1*.

The thematic analysis for subthemes with sample representativeness below 50% can be found under “Thematic analysis Study 2” in the Supplementary Material.

**Broad Theme:** Meaningfulness of strength changes in 6 weeks.

**Subtheme:** Any change in 5 weeks may be meaningful.

##### PL Athletes

Fifty percent of PL athletes expressed that any change in strength over 6 weeks is meaningful. They mentioned that in their “*eyes any sort of progress no matter how big or small means that whatever you're doing is working”* and that “*for me personally any change would be meaningful in just 6 weeks*.” Some even expanded further and attempted to quantify what “any change” may look like. They stated that “*any change [would be meaningful] because for some lifts, like, you know, you're squatting the same weight and training five days a week for a year. So, if you get 2.5 kilos or five kilos, it's like ‘Hallelujah! I made some progress!”’* Some others mentioned how their years of PL experience directly affect the rate of progress and thus any change would be meaningful. They expressed that “*because I've been in the game for so long and, this why my numbers will be very different from other people in 6 weeks, there would be a good chance I might not have any gain. But if I was five pounds stronger, two and a half kilos stronger in the deadlift, and the squat, I would consider that very successful and in the bench if I simply did the weight easier [that would be meaningful].”*

##### PL Coaches

Similarly to the PL athletes, 50% of PL coaches expressed that any change in strength over 6 weeks may be meaningful. They mentioned that “*after a high level, usually anything is meaningful*” and that “*anything above where they started [would be meaningful]*.” Others expressed how PL athletes would usually not train in 6 weeks blocks but “*if it's an advanced lifter, you know, you've done well if you've gained maybe 2.5 [kg] on the squat until point five on the deadlift, and you've done really, really, it's been an incredibly, incredibly successful block, even though truthfully, you would never get that from a block because you're probably looking at a more long term periodized approach to it.”* Similarly to the other PL coaches and some of the PL athletes, some coaches expressed that any change would be meaningful for non-beginners, saying that “*any change in a 6 weeks period would be pretty good for an intermediate or, or advanced level powerlifter.”* Some others mentioned how “*If they gain, you know, any amount of strength in 6 weeks, I'm going to give that a good block because there are plenty of blocks that don't go well at all and they can get worse”* briefly touching on how training outcomes may vary and increases in strength are not always apparent during training blocks.

**Broad Theme:** Factors affecting the magnitude of meaningfulness of strength changes in 6 weeks.

**Subtheme:***Strength level and experience of the athlete*.

##### PL Athletes

Twenty percent of PL athletes expressed that the strength level of the athlete will affect the magnitude of meaningfulness of strength changes in 6 weeks. A PL athlete expressed how they “*honestly think it would depend on the level of the person. A beginner lifter, you know, or an advanced lifter something significant could be as like as little as one kilo increase. You know that for me [is] significant? I think that if it's a beginner or an intermediate, a kilo, I don't know, wouldn't be as significant*.” Another PL athlete expanded on the same subtheme saying “*I think the more advanced someone is the more the meaning if you know what I mean. Like, for someone who has been training for years, even the slightest increase may mean a ton. Now for some beginners 10–20 kilos may not be that much but for someone with experience that sort of change could mean the world literally.”*

##### PL Coaches

Seventy two percent of PL coaches expressed that the strength level of the athlete will affect the magnitude of meaningfulness of strength changes in 6 weeks. Some coaches expressed how meaningfulness “*would largely depend on I would say, their current strength level and experience level more than [sic] any other factors. Obviously an athlete who is already very strong is difficult to make stronger. Not impossible by any means, but the training requires a little more creativity and sometimes it will take a few different rounds of training to establish what is the working protocol for that athlete, not one approach works for all athletes. There is a learning component to creating effective programming. So, those are the biggest factors I think, any other factors are a bit too random with regards to gender and things like that.”* A PL coach expanded on their answer providing insight on how novices may be able to make very large increases in 1RM strength over a short period of time in comparison to stronger, more experienced athletes, stating “*If you take an absolute beginner, Jesus man, you know, we can in decent form and go on 6 weeks program, even, I mean 30–40 kilos on a squat is totally doable, but how much of that has to do with coordination and skill acquisition vs., you know, so, vs. actual kind of, you know, force production gains.”* One further expanded on how meaningfulness of change will be directly affected by the athlete's training experience and strength level as “*When an athlete can squat 300 at 90 kg bodyweight, a 2.5% increase would be considered a lot, meaning that a meaningful increase could be far less, say 1%. For advanced lifters, 1–2.5% would be considered as meaningful.”*

#### Discussion

The results of study 2 not only aid the interpretation of studies 3 and 4, but can also help other researchers, coaches, and athletes assess when meaningful changes in the powerlifts have occurred. No previous study has explored what PL athletes and coaches regard as meaningful strength increases in 6 weeks. Indeed, studies utilising such elicitation methods are relatively uncommon in sport and exercise science (Steele et al., 2020). The PL athletes expressed that a PL total increase of 17.7 ± 12.1kg would be meaningful over a 6 weeks training period, which was similar to the 16.8 ± 16.3kg increase that the participants that were both athletes and coaches deemed meaningful. Similarly to the participants that were both athletes and coaches, participants who were only PL coaches expressed that a PL total increase of 16.6 ± 11.5kg would be regarded as meaningful over 6 weeks. Additionally, when looking at each powerlift individually, larger increases were needed for the SQ and DL in order to be regarded as meaningful in comparison to the BP (7.1 ± 5.1 and 8.1 ± 4.9 vs. 4.4 ± 3.3 kg, respectively). The difference in absolute changes may be due to the SQ and DL involving more and larger muscle groups than the BP and thus, allow heavier loads to be lifted; these between-lift differences may be smaller when assessed relatively as a percentage increase.

Somewhat in contrast to the survey responses, when looking at the interview responses of the elite PL athletes and experienced PL coaches, 50% expressed that any change in strength in 6 weeks may be meaningful. These responses can possibly be explained by the extremely high level of the PL athletes and coaches that were interviewed. A study by Latella et al. (2020) explored the long-term strength adaptations of powerlifters over 15 years by using data from the Powerlifting Australia database (www.powerliftingaustralia.com). They looked specifically at data from raw PL athletes, much like the sample used in this survey study. They found that since their first competition, male and female athletes gained ~0.15 ± 0.44 and 0.12 ± 0.69 kg per day respectively. They also found that for females, the extrapolated strength gain per year was 43.8 and 54.75 kg for males. Based on the above daily strength gains, male and female athletes included in the study by Latella et al. (2020) would gain ~6.3 and 5 kg in PL total strength in 6 weeks. These PL total increases are much lower than what was expressed by the participants of the survey, who expressed that a 17.5 ± 12.1 kg increase in PL total would be regarded as meaningful. Indeed, strength gains in PL athletes when exploring the open powerlifting dataset (https://www.openpowerlifting.org/) suggest that strength gains are relatively small and follow a linear-log relationship with time (Steele et al., 2021; see https://osf.io/preprints/sportrxiv/eq485/). Considering that strength gains become relatively smaller with training/competition age to the point of almost plateauing, this may help explain the responses of some of the PL athletes and coaches who expressed that any strength change in 6 weeks can be considered as meaningful, especially at the elite level. The rest of the responses were in the range of 12.5–25 kg for meaningful PL total increases with some respondents expressing that a >2% increase in any of the individual powerlifts would also be regarded as meaningful, largely agreeing with the respondents of the survey.

### Studies 3 and 4—The Effect of Different METD “Daily-Max” Protocols on 1RM Strength in Powerlifters

#### Materials and Methods

##### Design and Approach to the Problem

Studies 3 and 4 manipulated training dose to explore the effect of low volume, “daily max” training on 1RM strength in intermediate-advanced PL athletes. Study 3 compared a group following a protocol consisting of “daily max” high load single repetitions at RPE 9-9.5 versus a group following the same protocol with the addition of 2 “back-off” sets of three repetitions at 80% of the load used for the “daily max” single repetitions. “Daily max” refers to a near-maximal single repetition. A “daily max” single repetition can often be thought of as a powerlifter's “daily” 1RM which often differs from their tested 1RM. Study 4 compared a “daily max + back-off sets” protocol to a protocol where participants performed “as-many-repetitions-as-possible” (AMRAP) using 70% 1RM until they reached an RPE of 9-9.5. AMRAP sets instruct the athlete to perform as many repetitions as possible until reaching momentary, volitional failure or a prescribed RPE value. Aside from being a training tool, the use of AMRAP sets can often serve as a performance test for PL athletes, allowing them to compare the amount of repetitions achieved with their previous AMRAP, thus gauging progress.

Using quasi-randomized trial designs, three different “daily max” training protocols were compared in two studies using competitive and non-competitive PL athletes. Each training protocol was performed over a training period covering a 6 weeks cycle with 1 week pre-training intervention and 1 week post-training intervention dedicated to 1RM strength testing. When including the 1RM testing weeks the total length of each study was 8 weeks. Approval by the relevant ethics committee at Solent University was obtained (Health, Exercise and Sport Science Ethics Committee reference *andrp2018*).

##### Participants

During the early planning stages, prior to the survey and semi-structured interview studies, sample size was determined based upon traditional a priori power analysis (using G^*^Power) to ensure sufficient power to detect at least a large within-group effect in highly trained participants based upon thresholds from Rhea (2004). This suggested at least eight participants per group were required. We also considered what would be required for detecting a large between-group effect in an analysis of covariance design with baseline covariate adjustment for a large effect which suggested at least 26 per group. However, following the initial calculations, sample size was re-determined based on resource constraints and thus, was convenience-based and justified based on feasibility expectations given the authors' knowledge of the accessibility of the sample population (Lakens, 2021). Thus, we tried to recruit as many PL athletes as possible over the period of the lead author's PhD, which these studies were a part of. To aid this, recruitment was conducted over two sites: Southampton, UK and Patras, Greece. A total of 32 (16 participants per study, eight participants per group, four groups in total) male PL athletes, with at least two years of PL experience and at least four years of resistance training experience, were recruited. As research around PL training methods started receiving more attention by the scientific community in the last years, the concept of autoregulation in PL has been examined (Helms et al., 2018). Autoregulation using the RPE scale, based on repetitions in reserve (RIR), has been researched in the context of being utilized as a means of self-selecting training-loads. Autoregulation is a very common concept among PL athletes as it allows them to quantify their effort and appropriately select loads based on their readiness, rather than following a prescribed load and repetition scheme based on % 1RM (Helms et al., 2018). All participants were required to be raw PL athletes, have at least one year of RPE-based training experience using the RIR scale, and to not have followed any low-volume “daily max” training in the 12 weeks preceding the training intervention. Unfortunately, training facility closures due to the COVID-19 pandemic resulted in seven participants having to stop training while participating in study 4 and thus 25 participants remained for data analysis.

##### Group Assignment

The group assignment process was quasi-randomized. Justification for this was in part due to the desire to try to approximately match strength levels between groups given the small samples used and the potential for baseline imbalances in a factor prognostic likely to influence the outcomes. However, it was also due to knowledge of the population being recruited and that there is, in our experience, reluctance to take part in studies where training to be performed will be completely randomly allocated, even if only for short periods. Thus, we adopted an approach that we felt balanced these concerns. Participants were classified based on their strength level by a second researcher and PL coach working with the athletes and were then randomly assigned to a group by the lead researcher who was blinded regarding the participants' identity. Participants' strength was determined by their Wilks score. The classifications used were the following: participants with a Wilks score from 300 to 340 were classified as class 1, participants with a Wilks score of 350–400 were classified as class 2, participants with a Wilks score of 410–450 were classified as class 3, participants with a Wilks score of 460–500 were classified as class 4 and lastly, participants with a Wilks score of 510–550 were classified as class 5. The final sample sizes and characteristics of participants in each group can be seen in Table 8.

**Table 8:** Study 3 and 4 participant characteristics.

	**Age**	**Height** **(cm)**	**Weight** **(kg)**	**Training experience** **(years)**	**PL experience** **(years)**	**Competition experience** **(number of competitions)**	**Pre SQ 1RM** **(kg)**	**Pre BP 1RM** **(kg)**	**Pre DL 1RM** **(kg)**	**Pre total** **(kg)**	**Wilks**
**Study 3**											
MAX (*n* = 8)	28.8 ± 5.5	174.8 ± 2.8	85.8 ± 13.5	8.3 ± 6.5	4.3 ± 3.7	6 ± 13.8	182.5 ± 36.3	126.5 ± 18.5	215 ± 31.8	524 ± 71.4	347 ± 31.4
MAX+boff (*n* = 8)	28 ± 6.8	175.1 ± 3.8	88.1 ± 6.8	8.6 ± 6.6	4.5 ± 3.2	4 ± 5.6	196.8 ± 29.9	122.1 ± 17.5	216.2 ± 44.1	535 ± 86.8	347.2 ± 56.5
**Study 4**											
MAX+boff (*n* = 5)	28.8 ± 4.6	179 ± 6.2	94.8 ± 17.3	6.2 ± 3.4	2.7 ± 1.7	2.4 ± 2.3	168.5 ± 29.7	121 ± 24.6	202 ± 27.9	491.6 ± 75.8	310.4 ± 27.1
AMRAP (*n* = 4)	26.5 ± 3	169.2 ± 8.2	81 ± 11	6.2 ± 2.2	3.5 ± 1.9	3.2 ± 2.6	167.5 ± 26.3	110.6 ± 22.0	193.7 ± 24.3	471.8 ± 66	321.8 ± 23.7

*Results are mean ± SD, AMRAP refers to ‘As Many Repetitions As Possible’, MAX+boff refers to “daily max + back-off sets.”*

##### Testing

All participants underwent 1RM testing 7 days prior and 5 days after the 6-week training intervention. The 1RM testing was performed in a competition-like setting, requiring participants to test their SQ, BP, and DL 1RM all on the same day, with three attempts allowed for each powerlift. Participants were allowed to decrease load if they missed on a first attempt and testing sessions were overseen by an experienced investigator who, when necessary, aided in attempt selection. Participants were required to warm-up by gradually increasing the load and decreasing repetitions as they approached a load ~10% lighter than their desired first attempt. Participants had ~5 min of rest between attempts and ~15–20 min of rest between each powerlift. This testing approach, which is similar to that employed by Androulakis-Korakakis et al. (2018) who used an actual competition, was intended to lend ecological validity to the outcome measures.

##### Training

After the testing week, participants began their assigned training intervention over a 6-week period. Training sessions were completed at different training facilities in Southampton, UK and Patras, Greece. Most sessions were overseen by the lead researcher with some being overseen by one of the other researchers. In the case where a session could not be overseen by any researcher (for logistic reasons due to scheduling issues), participants were required to film all their working sets and return these as evidence to the lead researcher. In studies 3 and 4, two intervention groups were examined: “daily max” (MAX) and “daily max + back off sets” (MAX+boff). Albeit not directly examining the effect of AMRAP sets on 1RM strength using moderate loads, the results of the literature review chapter demonstrated that significant 1RM increases may be possible with higher repetitions sets utilizing loads as low as 70% 1RM. After preliminary examination of the results from study 3, we opted in study 4 to take forward and collect additional data for the MAX+boff intervention again, and also to include an additional group based on previous meta-analysis exploring the METD in trained participants (Androulakis-Korakakis et al., 2019): the AMRAP group. All three interventions were performed with a 2-3-1 SQ-BP-DL frequency, performing the SQ on days one and three, the BP on all three days and the DL on day two. The training days of all training protocols were Monday (day 1), Wednesday (day 2), and Friday (day 3). Further, no additional sets or exercises were performed during the 6 weeks training intervention by any group. The warm-up procedure for all the participants of the MAX and MAX+boff groups was similar to the warm-up during the testing procedure as they gradually increased the load and decreased repetitions as they approached their “daily max” set. The AMRAP group performed sets of five repetitions with 40, 50, and 60% of their 1RM before their main set with a load corresponding to 70% 1RM which was performed to an RPE 9-9.5.

##### MAX Group

The training protocol that the MAX group followed consisted of one set of a single repetition at RPE 9-9.5 for three training sessions per week. The PL athletes self-selected a load they believed enabled them to reach an RPE of 9-9.5, meaning they could either do one more repetition or they could not do more reps but could possibly do slightly more load (Helms et al., 2016).

##### MAX+boff Group

The training protocol that the MAX+boff group followed was the same as the MAX group, but with the addition of the performance of two “back-off” sets of three repetitions at 80% of the load they had lifted for their “daily max” single repetition.

##### AMRAP Group

The training protocol that the AMRAP group followed consisted of one AMRAP set using 70% 1RM until RPE 9-9.5 was reached. The PL athletes performed as many repetitions as they believed enabled them to reach an RPE of 9-9.5.

##### RPE

Participants' RPE scores (Helms et al., 2016) were recorded for all their working sets, including back-off sets, during all the training sessions of the intervention.

##### Muscle Soreness

Muscle soreness was assessed using the six point Likert scale of muscle soreness from Vickers (2001) 24 h after each training session. Participants were asked to note their muscle soreness score on a spreadsheet that was provided to them at the start of the study. The muscle soreness scale can be found in the Supplementary Material.

##### Enjoyment, Adherence, and Perceived Effectiveness

The effect of all protocols on training enjoyment, adherence, and perceived effectiveness were assessed using a questionnaire similar to that used by Androulakis-Korakakis et al. (2018). The questionnaire can be found in the Supplementary Material.

##### Data Analysis

These studies were not pre-registered and further, given the nature of the area and the constraints described, we have considered all analysis and results to be exploratory in nature. Despite initially considering the application of frequentist Neyman-Pearson null hypothesis significance testing (which also informed our initial a priori power analysis), we ultimately decided that this work was not yet at a stage to permit such testing. We further considered the extensive criticism directed at the dichotomisation of the existence of effects utilizing such a framework (Amrhein et al., 2019a; McShane et al., 2019). Thus, we opted to take an estimation based approach instead (Gardner and Altman, 1986; Cumming, 2013), based within a Bayesian framework (Kruschke and Liddell, 2017) which has been suggested as a worthwhile approach in sport science where samples and effects are often both small (Mengersen et al., 2016). For all analyses effect estimates and their precision, along with conclusions based upon them, were interpreted continuously and probabilistically, considering data quality, plausibility of effect, and previous literature, all within the context of each outcome (Amrhein et al., 2019b; McShane et al., 2019). We adopted the Bayesian approach of determining a “ROPE” (Region of Practical Equivalence); we utilised the survey data from study 2 to determine the range values that participants considered as meaningful changes in outcomes (we utilise the terminology “ROPE” throughout but note that strictly speaking the manner in which we use this is not exactly equivalent to the traditional threshold based approach, nor are we using it as a band of effects considered to be practically equivalent to a null effect). In addition to this “subjective” ROPE, we also include analysis in the Supplementary Materials using an “objective” ROPE which was determined from modelling of the open powerlifting dataset (https://www.openpowerlifting.org/) to determine the increase in SQ, BP, DL, and PL total bests to result in an increase of one position in yearly rankings within weight classes. Lastly, we note that any inferential statistics from the analyses of datasets generated from the participants sampled should be treated as highly unstable local descriptions of the relations between our model assumptions and data to acknowledge the inherent uncertainty in drawing generalised inferences from single samples (Amrhein et al., 2019b).

All analysis was conducted in R (v 4.0.2; R Core Team, https://www.r-project.org/) and all data and code are available in the Supplementary Materials (https://osf.io/fm2bh/). Descriptive statistics (means and standard deviations) were calculated across sessions and are reported for SQ, BP, and DL for RPE across sets, next-day muscle soreness and also for training enjoyment measures. Bayesian regression models described below were all fit using the “brms” package (Bürkner, 2017, 2018) with posterior draws taken using “tidybayes” (Kay, 2020) and “emmeans” (Lenth, 2020). All data visualisations were made using “ggplot2” (Wickham, 2016), and “patchwork” (Pedersen, 2020). Within the visualisations we note the model specification in Pinheiro-Bates-modified Wilkinson-Rogers notation (Wilkinson and Rogers, 1973; Pinheiro and Bates, 2001) for reference.

First, for determination of our “ROPE,” we fit a simple intercept only model,

$$\begin{array}{l} Y_{i}=a+e_{i} \end{array}$$

to the responses to the survey data described in study 2 for each outcome (SQ, BP, DL, and PL total). For each of the four Monte Carlo Markov Chains 1,000 warmup and 1,000 sampling iterations were used. Uninformed default priors were used for this model. Draws were taken from the posterior distribution (*n* = 4,000) for the model intercept term in order to construct a probability density function for the ROPE. This was in order to incorporate the uncertainty in our ROPE into our modelling approach, i.e., that different individuals had different responses as to what they considered a meaningful change.

We then conducted analysis of both intervention arms within both studies. For the analysis within each study we fit an analysis of covariance (ANCOVA) model on the change score in outcomes (i.e., post minus pre scores) as the dependent variable, and with adjustment for the pre scores as a covariate,

$$\begin{array}{l} Y_{i}^{Change\;score}=\left(\beta_{00}+u_{0i}\right)+{\beta_{10}Y}_{i}^{Pre\;score}+\beta_{20}Group_{i}+e_{i} \end{array}$$

In study 3, we set an informed prior on the intercept which was coded to represent the mean for the MAX group. Our informed prior was set based upon the means and standard deviations for pre-peri change scores reported using the “daily max” approach piloted by Androulakis-Korakakis et al. (2018). In study 4, we set an informed prior on the intercept which was coded to represent the mean for the MAX+boff group. In this case the informed prior was set based upon the means and standard deviations for the change scores of the MAX+boff group in study 3. We similarly used four Monte Carlo Markov Chains with 1,000 warmup and 1,000 sampling iterations. Draws were taken from the posterior distributions (*n* = 4,000) for the estimated marginal means of each group within each study in order to construct a probability density function for each. We then considered the effects in the following probabilistic frames. First we calculated the mode and the 95% highest density interval (HDI) from the posterior probability density functions for each group effect estimate. These gave us the most probable value of the parameter, in addition to the range over which there was a 95% probability that the parameter lay within. Next we assessed discrimination between each group and the ROPE using the area under the receiver operating characteristic curve (AUC). This was performed using the “ROCit” package (Khan and Brandenburger, 2020). Because AUC is an indicator of discriminability it can be interpreted as the probability that a randomly sampled intervention group effect draw is superior to a randomly sampled ROPE draw. Lastly, we looked at identifiability as the overlap (using a custom built function; see analysis code) in each groups distribution with the ROPE distribution wherein the overlap can be interpreted as the probability that a randomly sampled intervention group effect draw is “equivalent” to a randomly sampled ROPE draw. Thus, we had point estimates of effect magnitudes with their uncertainty, a probability that a given intervention group effect was larger than the ROPE, and also a probability that a given intervention group effect was “equivalent” to the ROPE.

As a final analysis, and given both the current scarcity of data on this type of training and the small group sizes in these studies, we examined a combined model examining the effects of the three “daily max” groups (i.e., MAX group from study 3, and the MAX+boff groups from studies 3 and 4) similar to an internal “meta-analysis” (Goh et al., 2016). This was a multilevel extension of the ANCOVA models employed within studies yet with the removal of the condition coefficient. The resulting mixed-effects model, in Pinheiro-Bates-modified Wilkinson-Rogers notation (Wilkinson and Rogers, 1973; Pinheiro and Bates, 2001) for brevity's sake, was,

$$\begin{array}{l} Change\_Score\sim Pre\_Score+\left(1|Study/Group/Participant\right) \end{array}$$

Thus, this model was intended to provide an overall analysis of “daily max” type training broadly speaking. As with the within study models, we calculated mode and 95% HDI, AUC, and overlap with the ROPE.

#### Results

**Figure 1:**
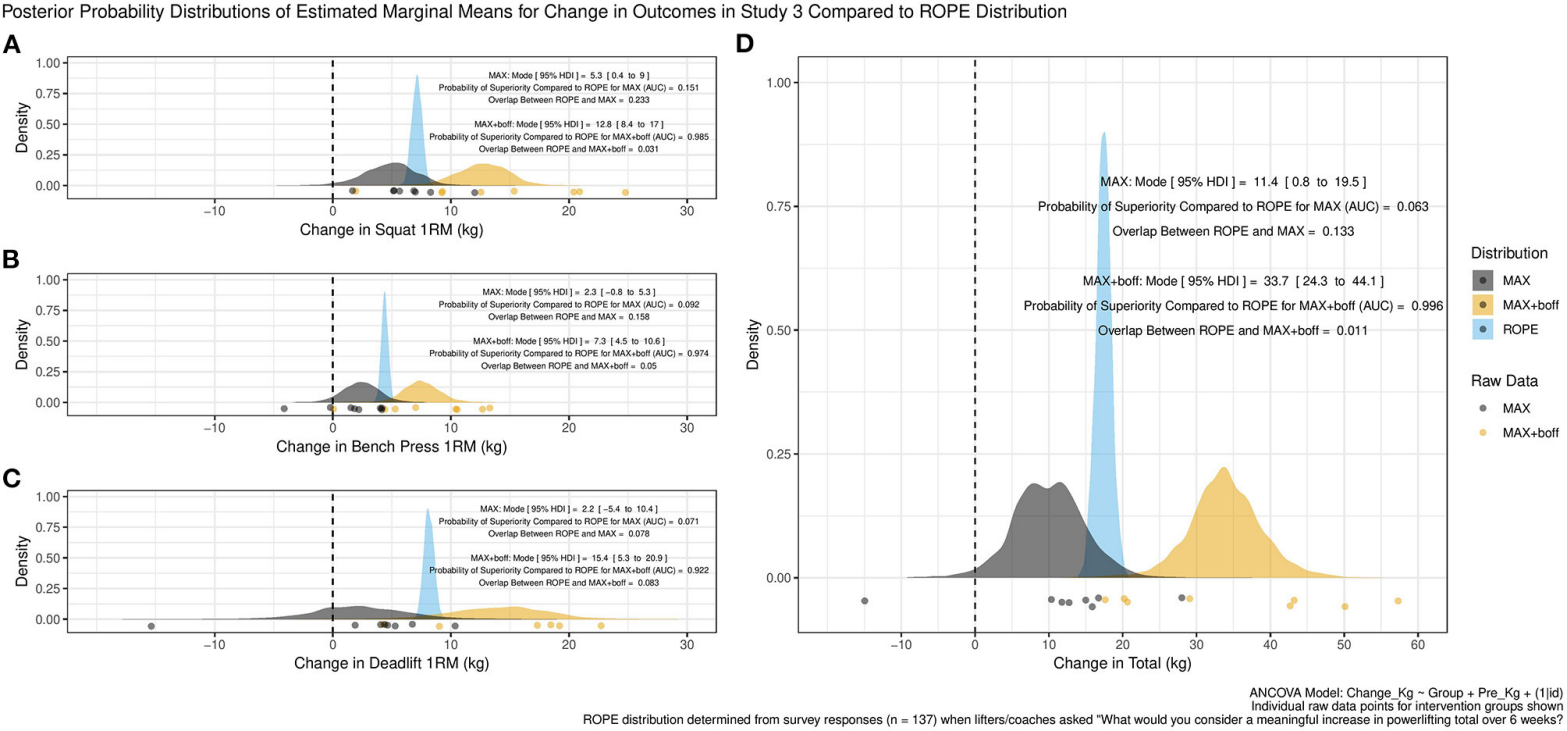
Study 3 posterior probability distributions of estimated marginal means for changes in outcomes compared to “Region of Practical Equivalence” (ROPE) distribution for the **(A)** squat, **(B)** bench press, **(C)** deadlift, and **(D)** powerlifting total. AUC is the receiver operating characteristic area under the curve.

Within study 3, analysis suggested that for SQ and BP both MAX and MAX+boff conditions likely produced increases in 1RM strength (Figures 1A,B), though DL estimates were far less certain as evidenced by the wider 95%HDI in both groups (Figure 1C). The MAX+boff group appeared to produce increases in DL 1RM strength but, with a sizable portion of the posterior probability distribution encompassing decreases, the MAX group effects were less clearly positive. For both groups, PL total appeared to increase (Figure 1D). The pattern of results are largely similar across the SQ, BP, and DL, and so focusing on the PL total outcome: the MAX group had a posterior probability distribution modal increase of 11.4 kg (95% HDI, 0.8–19.5 kg) with the AUC suggesting only a 6.3% probability of increases greater than the ROPE and 13.3% probability of increases within the ROPE, while the MAX+boff had a posterior probability distribution modal increase of 33.7 kg (95% HDI, 24.3–44.1 kg) with the AUC suggesting a 99.6% probability of increases greater than the ROPE and 1.1% probability of increases within the ROP.

**Figure 2:**
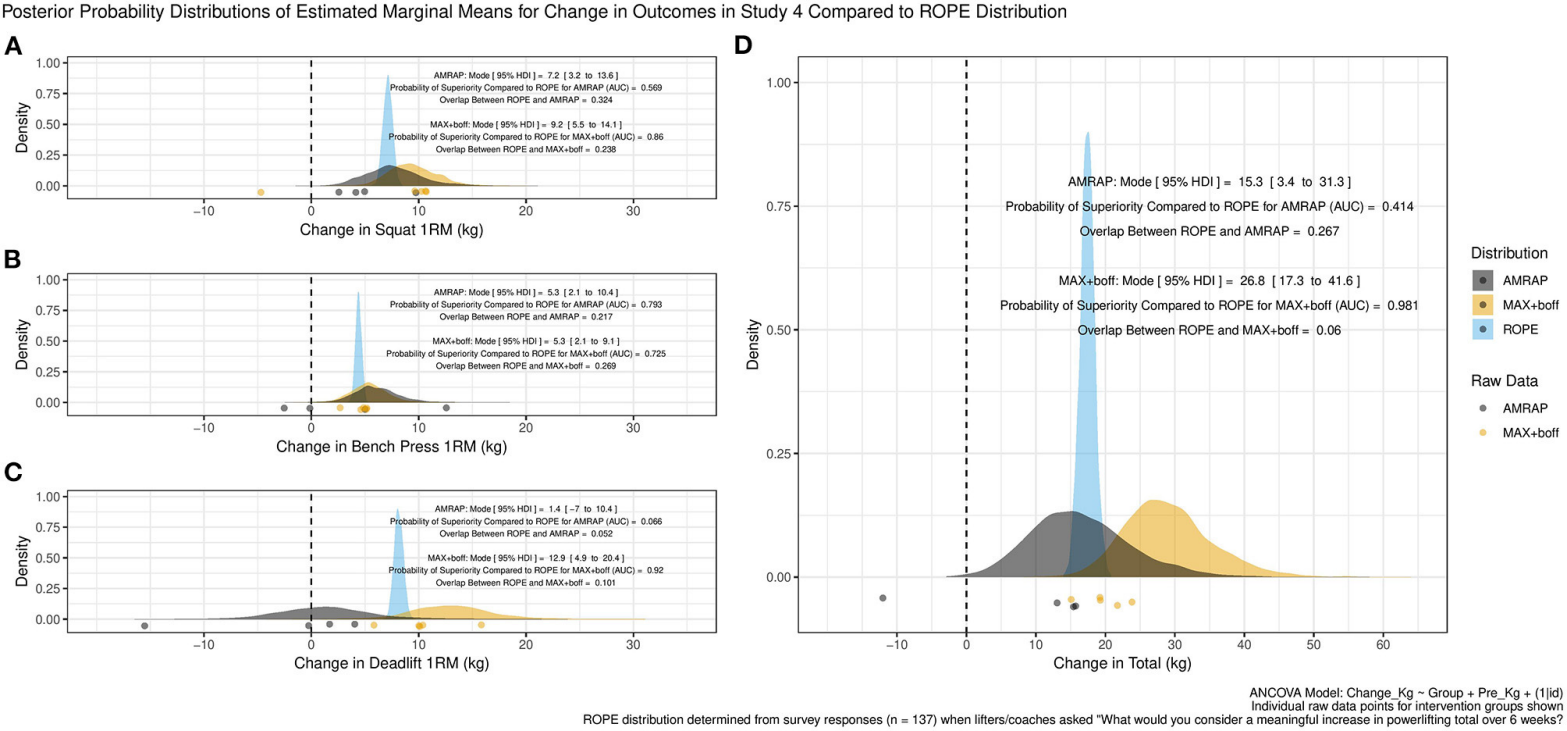
Study 4 posterior probability distributions of estimated marginal means for changes in outcomes compared to “Region of Practical Equivalence” (ROPE) distribution for the **(A)** squat, **(B)** bench press, **(C)** deadlift, and **(D)** powerlifting total. AUC is the receiver operating characteristic area under the curve.

Study 4 showed a similar pattern of results with respect to the SQ, BP, and DL, with the latter exhibiting far less precision with respect to estimates of change. Again, analysis suggested that for SQ and BP both MAX+boff and AMRAP conditions likely produced increases in 1RM strength (Figures 2A,B) which appeared far more similar in distribution than the comparison of MAX and MAX+boff effects in study 3. Further, while the MAX+boff group again appeared to produce increases in DL 1RM strength, the AMRAP group exhibited a sizable portion of the posterior probability distribution encompassing decreases with effects appearing far less clearly positive (Figure 2C). However, both groups' PL total appeared to increase (Figure 2D). Within study 4, and with incorporation of the prior for the MAX+boff from study 3, the MAX+boff group in this study demonstrated lower estimates of PL total change having a posterior probability distribution modal increase of 26.8 kg (95% HDI, 17.3–41.6 kg) with the AUC suggesting only a slightly reduced but still reasonably high 98.1% probability of increases greater than the ROPE and 6% probability of increases within the ROPE. The AMRAP group exhibited a posterior probability distribution modal increase of 15.3 kg (95% HDI, 3.4–31.3 kg) with the AUC suggesting a 41.4% probability of increases greater than the ROPE and 26.7% probability of increases within the ROPE.

**Figure 3:**
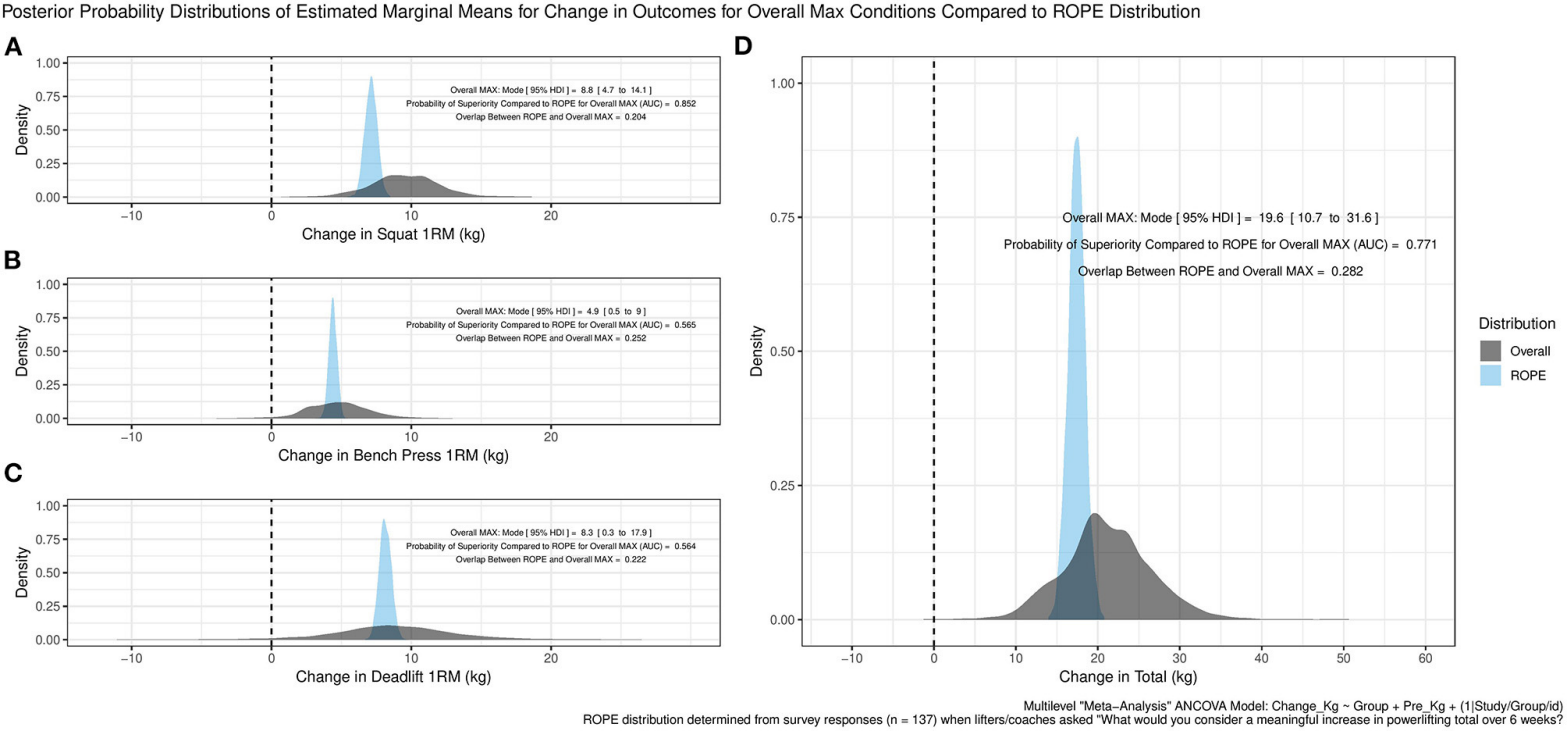
“Daily Max” groups of Study 3 and Study 4 posterior probability distributions of estimated marginal means for changes in outcomes compared to “Region of Practical Equivalence” (ROPE) distribution for the **(A)** squat, **(B)** bench press, **(C)** deadlift, and **(D)** powerlifting total. AUC is the receiver operating characteristic area under the curve.

Finally, the internal “meta-analysis” across the combined “daily max” groups (MAX and both MAX+boff groups) suggested likely positive increases across SQ, BP, and DL (Figures 3A–C). PL total change exhibited a posterior probability distribution modal increase of 19.6 kg (95% HDI, 10.7–31.6 kg) with the AUC suggesting a reasonably high 77.1% probability of increases greater than the ROPE and 28.2% probability of increases within the ROPE (Figure 3D).

Across all studies and groups, the results were broadly similar when compared to the “objective” ROPE (see Supplementary Materials). This was primarily due to the similarity in ROPE distributions for “objective” and “subjective” [e.g., for PL total modal increases (95% HDI) were: “objective” = 16.1 kg (14.3–18.5 kg); “subjective” = 17.5 kg (15.3–19.4 kg); https://osf.io/anwku/].

Descriptive data suggested that across groups, average RPEs attained during main working sets were similar and met the levels prescribed (i.e., 9-9.5 pts). Further, back off sets for the MAX+boff groups were lower with respect to RPE (~6–7). Muscle soreness levels were relatively low across all groups, though slightly higher by ~1 for the AMRAP group. Table 9 reports the means and standard deviations for these outcomes. The participants' responses to the post-intervention training questionnaire can be found in Supplementary Material.

**Table 9:** Studies 3 and 4 participant training characteristics.

	**Average RPE SQ main set**	**Average RPE BP main set**	**Average RPE DL main set**	**Average RPE SQ back-off set**	**Average RPE BP back-off set**	**Average RPE DL back-off set**	**Muscle soreness (/5)**
**Study 3**							
MAX	9.2 ± 0.1	9.3 ± 0.2	9.2 ± 0.1	N/A	N/A	N/A	0.7 ± 0.3
MAX+boff	9.2 ± 0.1	9.1 ± 0.2	9.2 ± 0.2	6.5 ± 1.4	6.6 ± 1.5	6.6 ± 1.8	1.1 ± 0.6
**Study 4**							
MAX+boff	9.4 ± 0.1	9.1 ± 0.2	9.2 ± 0.2	6.7 ± 0.6	6.7 ± 0.6	6.2 ± 1.4	1 ± 0.7
AMRAP	9.2 ± 0.2	9.2 ± 0.3	9.1 ± 0.2	N/A	N/A	N/A	2.1 ± 0.3
**AMRAP Repetitions**	**AMRAP Reps SQ**	**AMRAP Reps BP**	**AMRAP Reps DL**				
	13.1 ± 1.9	16.7 ± 2	11.5 ± 2				

*Results are mean ± SD*.

#### Discussion

Studies 3 and 4 explored variations of “daily max” type training, such as employed by Androulakis-Korakakis et al. (2018) and also with the inclusion of additional volume through addition of “back off” sets, as well as an AMRAP (“as many reps as possible”) type approach based off a previous review exploring the METD in trained persons (Androulakis-Korakakis et al., 2019). Due to the inherent difficulty of performing intervention studies in PL athletes, it was anticipated that sample sizes would be low, and thus study 2 was conducted to aid in interpretation of the meaningfulness of effects in a probabilistic Bayesian inference manner. In study 3, though the MAX group appeared likely to produce increases in PL total, these had a low probability of either meeting (13.3%) or exceeding (6.3%) a meaningful change, according to the criteria for “meaningful change” established in study 2. In study 4, the AMRAP group exhibited a 41.4% chance of exceeding a meaningful change with a 26% chance of meeting a meaningful change. However, in both study 3 and 4, the MAX+boff group experienced strength increases that had a high probability (study 3 99.6%, updated to 98% in study 4) of exceeding what PL coaches and athletes considered a meaningful change.

As mentioned above, the pilot study that employed the exact same protocol as the MAX group by Androulakis-Korakakis et al. (2018) found that 4 out of 5 PL athletes managed to increase their peri-training intervention total by 15, 25, 20, and 11 kg, respectively. However, only 2 of those 5 PL athletes experienced potentially meaningful increases during competition, increasing their PL total by 20 and 25 kg, while the other three participants experienced performance decreases. Despite “practicing the test” by training with near maximal loads (Dankel et al., 2017, 2020; Mattocks et al., 2017; Buckner et al., 2021), the extremely low training volume used in the MAX group may not be enough to allow for experienced PL athletes to make meaningful progress in 6 weeks and may potentially lead to deleterious effects after longer periods of training (though these decreases may also have been due to attempt selection on the day of competition). It is important to note that results from study 3 suggest training with a few heavy single repetitions per week may still yield strength increases, yet the probability that these would be enough to be considered meaningful is low. However, in some cases such changes may be deemed meaningful if we consider that some interview respondents noted that any change in 1RM strength in experienced athletes may be important.

After inclusion of study 3's results as prior, study 4 revealed a final updated probability of 6% that MAX+boff would produce a meaningful change, and 98% probability that this change would in fact exceed what is considered meaningful. This suggests the addition of only two sets consisting of three repetitions with ~2–4 RIR following heavy single sets can lead to meaningful strength increases. Despite being a seemingly small addition, boff sets amounted to the MAX+boff group increasing their overall SQ, BP, and DL training volume by an additional 12, 18, and 6 weekly repetitions, respectively. When compared to the MAX group, the MAX+boff group performed 600% more volume. However, despite this, the MAX+boff intervention is still a relatively low training dose and may reflect the METD, especially when considering that traditional training approaches can represent ~1,000–1,800% more training volume than a “daily max” approach (Androulakis-Korakakis et al., 2018). Aside from a possibly greater overall stimulus, the added training volume from the back-off sets may have contributed to the participants' overall 1RM improvement by adding to the skill component of 1RM strength by providing extra practice of the powerlifts. Additionally, previous research examining the METD looked at the literature exploring the effects of single versus multiple sets has found that in certain cases, multiple sets per week can produce greater 1RM strength increases than just 1 set performed a few times per week (Androulakis-Korakakis et al., 2019); though the relationship between volume and strength is trivial to small (Ralston et al., 2017). It is important to note that most of the low volume groups included in the systematic review and meta-analysis by Androulakis-Korakakis et al. (2019), aside from the group in Ogasawara et al. (2013), were performing a similar, or higher, number of weekly repetitions for the SQ and BP when compared to the MAX+boff group in study 3 and 4. All the low volume groups identified in the systematic review and meta-analysis by Androulakis-Korakakis et al. (2019) achieved significant 1RM strength increases, indicating that suboptimal, but still meaningful 1RM strength increases are possible with lower volumes of training.

Indeed, as noted, study 4 compared a group using the same protocol as the MAX+boff group with a group following an AMRAP “daily max” approach following recommendations from Androulakis-Korakakis et al. (2019). This involved using a load corresponding to 70% of the participants 1RM for “as many repetitions as possible;” which, in this case, was not to momentary failure such as defined by Steele et al. (2017), but based on typical application by PL athletes, i.e., until reaching RPE 9-9.5 allowing one more repetition or slightly more load (Helms et al., 2016). The aim of including the AMRAP group was to explore the effect of moderate-load higher repetition “daily max” sets on 1RM strength, to understand whether PL athletes can utilize lighter loads when training with a METD approach. As previously mentioned, utilizing light to moderate loads can elicit significant strength increases, but when assessing strength *via* a 1RM test, heavier loads may result in greater strength increases (Lasevicius et al., 2018; Fisher et al., 2020; Schoenfeld et al., 2021). Interestingly, despite the AMRAP group performing more training volume than the MAX+boff group, they were unable to attain similar strength increases as the MAX+boff group. Indeed, similarly to the MAX group in study 3, the AMRAP group may still yield strength increases, yet the probability that these would be enough to be considered meaningful is low. Though again we note that, in some cases such changes may be deemed meaningful considering the responses of some interviewees.

The results of the AMRAP group may also be a result of the training proficiency of the participants. The participants were all PL athletes with years of RT experience as well as a few years of solely focusing on increasing their 1RM strength. Possibly due to their high level and specific training experience, the AMRAP group was not able to increase strength similarly to other “trained” populations who have been observed to progress using moderate to light loads for higher repetitions (Lasevicius et al., 2018). It is important to note that similarly to the results of study 4, the group in the Lasevicius et al. (2018) study that utilized heavier loads (80% 1RM) managed to increase their 1RM strength more than the lighter load conditions. Finally, the speculation that strength is better gained by lifters with more training experience when using higher percentages of 1RM is also supported by previous meta-analyses (Rhea et al., 2003; Peterson et al., 2005).

In addition to the strength gains, it is worth considering the practical application of the approaches explored by considering other self-report outcomes. In both studies (3 and 4), all groups scored relatively low in terms of muscle soreness with the highest soreness score being in the AMRAP group with a score of 2.1 ± 0.3 out of five. The relatively low training volumes across all groups may explain the low soreness scores as previous research investigating the effect of high-volume training protocols on muscle soreness has found significant increases in muscle soreness following high-volume muscle damaging resistance exercise (Sikorski et al., 2013).

### Study 5—Minimum Effective Training Dose Practices in Competitive Powerlifters: A Survey Study

#### Materials and Methods

##### Design and Approach to the Problem

This survey study aimed to explore and describe the METD for 1RM strength practices of competitive PL athletes as well as understand the prevalence of using a METD among PL athletes. It also aimed to explore the reasons why some PL athletes have not experimented with METD.

##### Participants

Participants for this study were recruited through personal networks and social media. As such, again, the sample size justification was resource constraint based (Lakens, 2021) in that we were constrained to the number of participants willing to respond to the survey. Prior to completing the survey, participants were asked to provide informed consent and were informed of the aims and risks of the survey. Participants were eligible to participate in the study if they had competed at the national level or higher in an IPF affiliated competition and were required to provide proof of their latest competition results which could be verified *via* openly available data (i.e., https://www.openpowerlifting.org/). Fifty eight PL athletes, 47 males and 11 females, took part in the study. The participants were all confirmed to be national level PL athletes with the male athletes having a 601.7 ± 110.2 kg PL total and the female athletes a 332.7 ± 56.7 kg PL total. Additional participant characteristics, including age, bodyweight, training experience, and PL experience can be found in Table 10.

**Table 10:** Study 5 participant characteristics.

**Characteristic**	**Male**	**Female**
N	47	11
Age (years)	29 ± 9.1	27.8 ± 9.6
Body Mass (kg)	93.7 ± 14.9	63.9 ± 9
Training Experience (years)	9.9 ± 7.2	5.4 ± 3.3
PL Experience (years)	4.2 ± 3.6	3.3 ± 1.5
SQ (kg)	210.1 ± 40.5	121.3 ± 23.2
BP (kg)	144.2 ± 36.4	69 ± 16.3
DL (kg)	247.3 ± 44.2	142.2 ± 24.7
PL Tota (kg)	601.7 ± 110.2	332.7 ± 56.7

*Results are mean ± SD*.

##### Procedures

A 59-item survey was constructed by the authors based upon their expertise of the area and populations. The survey included questions on the PL athlete's training and powerlifting experience, competition results, whether they had trained using a METD approach before and if not, why not, as well as multiple questions on the different training variables surrounding METD (loads used, sets, repetitions, additional exercises, etc.). Prior to answering any of the survey questions, participants were provided the following definition of the METD to help contextualise their responses: “*A ‘minimum effective dose’ training approach refers to training with the lowest possible training volume while still experiencing meaningful training increases.”* The survey questions can be found in the Supplementary Material. The survey was conducted on the online platform Typeform.com.

##### Analysis

As this survey was intended to be exploratory and descriptive, we focused on reporting descriptive statistics (means and standard deviations) and % of respondents for each item.

#### Results

A summary of the responses is provided in Table 11. Of the sample, only 36.2% (*n* = 21) PL athletes had experimented with a METD type approach before. The primary reasons for not experimenting with a METD approach appeared to be due to concerns relating to results/progress. The plurality of respondents noted that they wanted optimal results (43.2%), and also that they did not want to experiment and risk potential progress (29.7%). Other reasons related to non-training benefits perceived from higher volumes (21.6%) or that they enjoyed training with greater volumes (21.6%). Many had not thought of experimenting with a METD approach (32.4%) though the majority confirmed they would consider its use if there were more evidence regarding its effectiveness and utility (91.9%). For those that had experimented with a METD, though limited time was a key reason (47.6%), it appeared other reasons were related mainly to the management of fatigue (47.6%), injury (38.1%), and maintaining longevity in PL (33.3%). METD was primarily used when busy with factors outside training (61.9%), though also as a part of a competition preparation strategy (61.9%).

**Table 11:** METD responses.

**Reasons for not training with METD**	**Total (% of participants)**
I want optimal results	43.2% (*n* = 16)
I had not thought of training with a minimum effective dose approach before	32.4% (*n* = 12)
I do not want to experiment and risk potential progress	29.7% (*n* = 11)
I do not feel comfortable with doing less training volume than I am currently doing	21.6% (*n* = 8)
I enjoy spending as much time training as I can	21.6% (*n* = 8)
I get additional, non-strength related results/benefits from doing more training volume (e.g.,: improved sleep/mood)	21.6% (*n* = 8)
I train with a friend/partner who does not want to follow a minimum effective dose training approach	2.7% (*n* = 1)
Other	10.8% (*n* = 4)
I would consider occasionally utilizing a minimum effective training dose approach if there was more evidence around its effectiveness and overall utility	91.9% (*n* = 34)
**Why do you use a minimum effective dose training approach?**	**Total (% of participants)**
Limited time available	47.6% (*n* = 10)
Reduce fatigue	47.6% (*n* = 10)
Injury management	38.1% (*n* = 8)
I enjoy training more with a minimum effective dose approach	33.3% (*n* = 7)
Longevity in Powerlifting	33.3% (*n* = 7)
I find it easier to progress using a minimum effective dose approach	28.6% (*n* = 6)
Low motivation to train	14.3% (*n* = 3)
I do not enjoy overreaching symptoms	9.5% (*n* = 2)
Other	19% (*n* = 4)
**When do you use a minimum effective training dose approach?**	**Total (% of participants)**
Busy periods due to exogenous factors (e.g.,: work, studies, family)	61.9% (*n* = 13)
Competition preparation	61.9% (*n* = 13)
Off-Season	42.9% (*n* = 9)
Deload	33.3% (*n* = 7)
Other	14.3% (*n* = 3)
**Length of METD Training**	**Mean (± SD)**
Consecutive weeks	9.1 ± 10.7
Total months in a year	4.1 ± 2.8
Training days per week	3.5 ± 0.7

Table 12 reports the training variables across exercises when employing a METD approach. Training variables across the SQ, BP, and DL were actually quite similar when employing a METD approach with perhaps the exception of volumes (BP>SQ>DL), and frequency which followed a somewhat typical ~2-3-1 SQ-BP-DL days per week.

**Table 12:** METD training variables.

	**Weekly training frequency** **(days)**	**Weekly working sets**	**Repetitions per working set**	**%1RM used for the working sets**	**RPE for the working sets**	**Accessory exercises per week**	**Weekly working sets for the accessory exercises**	**Repetitions per accessory exercise working set**	**RPE for the accessory exercise working sets**	**Strength change** **(kg)**	**Meaningfulness of strength change** **(1–5 Likert scale)**
SQ	1.8 ± 0.5	5.4 ± 1.6	3.5 ± 1.4	80.5 ± 8.5	7.8 ± 1.1	2.1 ± 1	4 ± 1.8	9.3 ± 2.2	7.6 ± 0.9	14.8 ± 11	3.3 ± 1
BP	2.5 ± 0.7	7.6 ± 3.2	4.1 ± 1.8	80.4 ± 6.5	8.1 ± 0.9	2.6 ± 1.2	7.3 ± 6.6	9.2 ± 2.9	7.7 ± 0.7	7.7 ± 8.3	2.9 ± 1.3
DL	1.1 ± 0.3	3.8 ± 1.3	3.6 ± 1.3	78.3 ± 8.8	7.7 ± 1.3	1.6 ± 1.1	4.2 ± 3.1	8.8 ± 2	7 ± 1.3	14.2 ± 15.9	3 ± 1.5

*Results are mean ± SD*.

#### Discussion

Study 5 surveyed the use of METD approaches in national level PL athletes and found that the majority (63.8%) had not experimented with such an approach and this appeared primarily due to concerns regarding results/progress. However, such fears may be related to a lack of understanding of the possible effectiveness and utility of a METD approach. Specifically, of those participants who had not used it previously, 92% expressed that they would consider occasionally utilizing a METD approach if there was more evidence supporting it. It is perhaps unsurprising that PL athletes focus on optimization, as even minimal gains in performance may sometimes translate to better placing in competition (Ferland et al., 2020) and as found in study 2, some athletes and coaches consider any change to be meaningful. Just over 20% of PL athletes mentioned that they had not experimented with a METD approach before as they did not feel comfortable doing less training volume than they were currently doing. This perhaps suggests that there is at least some perception that greater training volumes are of benefit, though as noted the relationship between volume and strength is relatively small to trivial (Ralston et al., 2017) and load may be more important to maximal strength outcomes (Schoenfeld et al., 2019). The PL athletes' responses may be due to the uncertain relationship between training volume and maximal strength and the lack of research on METD training in strength athletes (Androulakis-Korakakis et al., 2019). It is important to note that a similar proportion (21.6%) of PL athletes also expressed that they have not experimented with a METD approach before as they believe they get additional, non-strength related benefits from more training volume (e.g.,: improved sleep and mood), as well as enjoying training for more time. Androulakis-Korakakis et al. (2018) also asked participants to rate the enjoyability of their protocols, yet noted similar responses for both the “daily max” METD and the traditional periodised higher volume protocols. The responses of PL athletes in this survey may stem from the lack of experience with METD approaches when considering the results of Androulakis-Korakakis et al. (2018), and participants' responses in studies 3 and 4, which indicate that METD training may be relatively enjoyable. It may also be, as observed in the results of study 1, that PL athletes will only consider utilizing a minimum effective dose approach when time is limited or when they are “not feeling 100%” (presented in detail under “Thematic analysis Study 1” in the Supplementary Material).

Limited time availability, fatigue and injury management, training enjoyment, as well as longevity in PL were some of the most common responses among the participants who had experience training with a METD approach. Previous research has highlighted how PL athletes will often use reduced volume, higher load training as a means of competition preparation as it allows them to reduce training fatigue while preserving, and improving performance (Pritchard et al., 2016; Travis et al., 2020). The ability to make meaningful progress, while spending less time training, may be what is contributing to the enjoyment of a METD training approach. It may also be that the low levels of muscle soreness, similarly to what was observed in studies 3 and 4, in conjunction with the lower fatigue from the relatively low training volume, also contribute to the overall enjoyment of a minimum training dose approach.

The responses regarding how a METD has been employed mirror the responses of the PL athletes and coaches of study 1 who also described the METD as a few heavy load sets per week performed with a relatively high intensity of effort. In terms of application, respondents noted they had used METD type approaches for 9.1 ± 10.7 consecutive weeks, for a total of 4.1 ± 2.8 months in a given year. Manipulation of training variables between SQ, BP, and DL with respect to loads (~80% 1RM), repetitions (~3–4 reps), RPE (~8), and also application of accessory lifts were relatively similar between exercises. However, weekly set volumes differed with BP (7.6 ± 3.2) being greatest, followed by SQ (5.4 ± 1.6), and then DL (3.8 ± 1.3); these may be influenced partly by the frequencies of training for lift which follow a somewhat typical ~2-3-1 days for the SQ-BP-DL each week. Albeit describing a minimum dose approach, the weekly sets performed for each powerlift are not all on the “low” side of weekly set volume. The meta-analysis on the effect of weekly set volume on strength gain by Ralston et al. (2017), classified anything over 10 weekly sets as high volume, with anything between 5 and 9 weekly sets categorized as medium and anything below 5 sets as low volume. The responses of the PL athletes placed them in the low-to-medium weekly set range for the SQ, the medium-to-high weekly set range for the BP and the low weekly set range for the DL. These results further relate to the interview responses of the PL athletes and coaches where they expressed that the BP may need additional training volume to progress when compared to the other two powerlifts. The results of study 5 also further highlight that METD may be slightly different for each powerlift; though future research is needed to explore the common frequency practices across the powerlifts.

As discussed above, the current evidence around METD shows that significant 1RM strength increases can occur by doing 2–3 working sets per week with a heavy load for 6–12 repetitions (Androulakis-Korakakis et al., 2019). Further, the results of studies 3 and 4 also show increases are possible with all the investigated iterations of METD. The results of this survey demonstrate that advanced PL athletes who have experimented with a METD approach utilize greater set volumes than what is found in the current literature on trained individuals, or that which has been investigated in studies 3 and 4 here. This may be due to strength athletes perceiving the need for more training volume to practice the skill of the powerlifts. The review by Androulakis-Korakakis et al. (2019) highlighted that there is currently no literature on the METD of highly trained strength athletes, something that may explain the discrepancy between the results of study 5 and the current body of research. The PL athletes expressed that they had experienced 1RM strength increases of 14.8 ± 11, 7.7 ± 8.3, and 14.2 ± 15.9 kg for the SQ, BP, and DL respectively over 6 weeks of METD training. The PL athletes also rated the meaningfulness of the strength changes using a 5 point Likert scale with a rating of 5 being “extremely meaningful” and a rating of 1 being “not meaningful at all.” The strength change meaningfulness ratings were 3.3 ± 1, 2.9 ± 1.3, and 3 ± 1.5 for the SQ, BP, and DL respectively. The strength increases reported for the SQ and BP are similar, albeit slightly higher than the estimated increases reported in the systematic review and meta-analysis by Androulakis-Korakakis et al. (2019) which reported a 1RM increase of 12.09 kg for the SQ and 8.25 kg increase for the BP. They do however, exceed descriptively the minimal meaningful changes reported by coaches and athletes in study 2, corroborating their meaningfulness.

### General Discussion

Overall, the studies conducted and reported here suggest that a METD may be successfully implemented for ~6–12 weeks and can potentially be useful for periods where time is limited e.g., deload and potentially pre-competition, and also to manage fatigue, injury, and enhance longevity in the sport. Triangulation of results from the interviews, surveys, and intervention studies suggest that a few heavy load sets per week per powerlift, ranging from 1 to 5 repetitions and sometimes including the use of “back-off” sets may be enough to meaningfully increase 1RM strength in PL athletes.

It is also important to note that the results of the training studies showed that 1RM strength can be maintained or slightly increased, albeit likely not meaningfully, with even fewer heavy load sets per week, sometimes as low as a single set of a single heavy load repetition per week. Spiering et al. (2021) recently reviewed the minimal dose of exercise needed to preserve endurance and strength over time and found that a key variable to *maintain* strength was load. Similarly to Spiering et al. (2021), when looking at the minimum dose required to increase, rather than maintain, strength over time, Androulakis-Korakakis et al. (2019) also found load as well as intensity of effort to be key variables in increasing strength with low volumes of training. In contrast, the meta-analysis by Ralston et al. (2017) suggested that higher training volumes (>10 sets per week) may optimize strength gains, yet increases with additional volume were relatively small to trivial. That said, large improvements in strength were seen even in the lowest weekly volume examined, suggesting that the vast majority of gains occur with relatively little volume. Based on the currently available evidence, the METD for powerlifters may be slightly higher than the METD for recreationally trained individuals. A recent study by Steele et al. (2021) analyzed the training data of 14,690 participants who had been training 1 time per week performing single sets to momentary failure on six exercises and found that the participants were able to make substantial strength increases for ~1 year. Further, several studies in untrained or recreationally trained participants have single 1RM lifts compared to more traditional resistance training produce similar improvements in 1RM strength (Dankel et al., 2017, 2020; Mattocks et al., 2017; Buckner et al., 2021). Yet, in the intervention studies 3 and 4 conducted and reported here, the addition of “back off” sets to such a “daily-max” protocol were required to increase the probability of producing meaningful strength gains.

When it comes to the METD, the insight provided by the PL coaches and athletes in study 1 demonstrate that the METD may be a concept applicable to PL athletes of all levels, ranging from beginners to elite. This is further supported by the responses of the PL athletes in study 5.

With respect to volume, frequency is also a variable that is typically manipulated by PL athletes and coaches and specifically with respect to the different powerlifts. The survey and interview data reported here corroborate a somewhat typical ~2-3-1 days for the SQ-BP-DL each week, and as such due to the similar within-session volumes, used a volume partitioning of BP>SQ>DL. It has been reported that anecdotally, some believe the DL may be more fatiguing than the SQ (Barnes et al., 2019), thus requiring less training volume, but a study by Barnes et al. (2019) found no differences in the neuromuscular and endocrine responses following acute SQ and DL training. Thus, it is not entirely clear whether such partitioning of volume and frequency is warranted. Studies 3 and 4 followed the typical 2-3-1 SQ-BP-DL frequency and interestingly, there was far less certainty in effect estimates for the DL. This may to some extent suggest that with very low doses due to both low session volumes and very low frequencies of training, the METD may produce more variable responses between individuals.

One of the main limitations of the intervention studies were the small sample sizes, as, notably, they limit the generalisability of the inferences we can make about their effects. That said, self-report responses from study 5 corroborate similar strength gains which were considered relatively meaningful by respondents. Another potential limitation, albeit minor, is that training pre-intervention was not adequately controlled for. Aside from the requirement of “no daily max training” close to the training intervention, there was no additional control on how the participants trained prior to the study, which could have impacted our results. Participants who were training with lighter loads and higher training volumes may have been able to experience greater 1RM strength increases than those who were not as “sensitized” to such a style of training. Nevertheless, a strength of the work conducted is the triangulation of methods from varying methodological perspectives enabling a richer exploration of the concept of the METD in PL.

As a final consideration, load may be a greater contributing injury risk factor than volume. Injury rates are higher in powerlifting than bodybuilding (Keogh and Winwood, 2017), injury prevalence is higher among powerlifters with stronger compared to weaker deadlift 1RMs, and injury rates during powerlifting competitions are higher than during training when factoring in the time athletes spend competing versus training (Spence et al., 2020). Therefore, while METD approaches may be useful for PL athletes in many circumstances, more research is needed to determine if adopting an approach where volume is substantially lower, but load is substantially higher (single-repetition sets at 9-9.5 RPE are equivalent to ~90–99% 1RM) modifies injury risks.

## Conclusions

PL athletes looking to train with a METD approach can do so by performing ~3–6 working sets of 1–5 repetitions each week, with these sets spread across 1–3 sessions per week per powerlift, using loads above 80% 1RM at an RPE of 7.5–9.5 for 6–12 weeks and expect to gain strength. PL athletes who wish to further minimize their time spent training can perform autoregulated single repetition sets at an RPE of 9-9.5 though they should expect that strength gains will be less. However, the addition of 2–3 back-off sets at ~80% of the single repetitions load, may produce greater gains over 6 weeks while following a 2-3-1 SQ-BP-DL training frequency. When utilizing accessory exercises in the context of METD, PL athletes typically utilize 1–3 accessory exercises per powerlift, at an RPE in the range of 7–9 and utilize a repetition range of ~6–10 repetitions.

PL athletes can utilize METD during periods of limited time available, deloads as well as a potential competition preparation tool. Further, doing so may be a useful strategy to manage fatigue, injury risk (among powerlifters who already train frequently with high loads and reduced volume), and thereby, enhance longevity in the sport.

## Data Availability Statement

The datasets presented in this study can be found in online repositories. The names of the repository/repositories and accession number(s) can be found at: https://osf.io/fm2bh/.

## Ethics Statement

The studies involving human participants were reviewed and approved by Solent University Health, Exercise, and Sport Science Ethics Committee. The patients/participants provided their written informed consent to participate in this study.

## Author Contributions

PA-K prepared the manuscript, performed the data analysis for studies 1, 2, and 5, and oversaw the majority of the data collection for all studies. JS revised the first draft of the manuscript and performed the data analysis for studies 3 and 4. NM oversaw and coordinated parts of the data collection for studies 3 and 4. EH revised the manuscript, assisted with the survey design for study 5, and the recruitment process for studies 1 and 2. JK, JL, and MW revised the manuscript and assisted with the survey design for study 5. JF and GN revised the manuscript. All authors contributed to the article and approved the submitted version.

## Conflict of Interest

GN was employed by Stronger By Science, LLC. The remaining authors declare that the research was conducted in the absence of any commercial or financial relationships that could be construed as a potential conflict of interest.

## Publisher's Note

All claims expressed in this article are solely those of the authors and do not necessarily represent those of their affiliated organizations, or those of the publisher, the editors and the reviewers. Any product that may be evaluated in this article, or claim that may be made by its manufacturer, is not guaranteed or endorsed by the publisher.
