# Supplementary material for: The Minimum Effective Training Dose Required for 1RM Strength in Powerlifters
Source: Front Sports Act Living. 2021 Aug 30;3:713655. doi: 10.3389/fspor.2021.713655 (PMC8435792; doi:10.3389/fspor.2021.713655)
Supplement: Supplementary file 1 [file Data_Sheet_1.DOCX]

**Supplementary Material**

**Tables
Study 1**

**Table A – Interview Questions (PL coaches)**

| What do you think about the concept of METD for 1RM strength in PL athletes? |
| --- |
| Based on your experience, what does METD look like? |
| What variables does METD depend on? |
| What level PL athletes would benefit most from such a training approach? |
| How would you implement a minimum effective training dose within a powerlifter’s training protocol? |
| For how long would you implement a minimum effective training dose approach? |

**Table B – Interview Questions (PL athletes)**

| What do you think about the concept of METD for 1RM strength in PL athletes? |
| --- |
| Based on your experience, what does METD look like for you? |
| What variables does METD depend on? |
| How would you implement a minimum effective training dose within your training program? |
| For how long would you implement a minimum effective training dose approach? |

**Study 2**

**Table C – Interview Questions (PL coaches & PL Athletes)**

| What would you consider to be a meaningful change in SQ, BP, DL and PL Total strength over a 6 week training cycle? |
| --- |
| What does a meaningful strength change depend on? |

**Table D – 6 week meaningful increases survey questions**

| **Question** | **Options (if applicable)** |
| --- | --- |
| Sex | Male / Female |
| Age (years) |  |
| Are you a PL athlete or coach | Athlete / Coach / Both |
| Training Experience (years) |  |
| Coaching Experience (if applicable / years) |  |
| Which level do you or your athletes compete in? | Regional/National/International |
| What would consider a meaningful increase in squat 1RM strength over 6 weeks (in kg)? |  |
| What would consider a meaningful increase in bench press 1RM strength over 6 weeks (in kg)? |  |
| What would consider a meaningful increase in deadlift 1RM strength over 6 weeks (in kg)? |  |
| What would consider a meaningful increase in powerlifting total over 6 weeks (in kg)? |  |

**Table E** – The RPE Scale Based on Repetitions in Reserve (Helms, 2016)

| Rating | Description |
| --- | --- |
| 10 | Could not do more reps or load |
| 9.5 | Could not do more reps, could slightly more load |
| 9 | Could do 1 more repetition |
| 8.5 | Could definitely do 1 more repetition, chance at 2 |
| 8 | Could do 2 more repetitions |
| 7.5 | Could definitely do 2 more repetitions, chance at 3 |
| 7 | Could do 3 more repetitions |
| 5-6 | Could do 4 to 6 more repetitions |
| 1-4 | Very light to light effort |

**Table F - 6-point Soreness Likert Scale**

| **Rating** | **Description** |
| --- | --- |
| **0** | A complete absence of soreness |
| **1** | A light pain felt only when touched / a vague ache |
| **2** | A moderate pain felt only when touched / a slight persistent pain |
| **3** | A light pain when walking up or down stairs |
| **4** | A light pain when walking on a flat surface / painful |
| **5** | A moderate pain, stiffness or weakness when walking / very painful |
| **6** | A severe pain that limits my ability to move |

**Table G – Enjoyment, adherence and perceived effectiveness questionnaire**

| **Questions** | **Options (if applicable)** |
| --- | --- |
| Which group did you belong to? | Daily MAX / Daily MAX + Back-offs / Daily MAX AMRAP |
| How enjoyable was the training protocol that you were assigned to? | 1-5 Likert scale |
| How easy was it to adhere to your training protocol? | 1-5 Likert scale |
| How manageable was the workload of your training sessions? | 1-5 Likert scale |
| How effective was your training protocol in covering your training needs? | 1-5 Likert scale |
| How likely are you to use the same or a similar training protocol in the future? | 1-5 Likert scale |
| How much did the training protocol impact current or past injuries? | 1-5 Likert scale |
| How likely are you to utilise such a training approach during a deload period? | 1-5 Likert scale |
| How likely are you to utilise such a training approach during a busy period with limited time available for training? | 1-5 Likert scale |
| How much did exogenous factors (stress, lack of sleep, personal issues) affect your performance during the training intervention? | 1-5 Likert scale |
| How accurately did you follow the protocol's guidelines (eg: RPE or load/reps assigned)? | 1-5 Likert scale |
| How confident were you at utilising the RPE scale during training sessions? | 1-5 Likert scale |
| How likely are you to utilise the same or a similar protocol during a strength gain phase? | 1-5 Likert scale |
| How effective was the protocol you followed in reducing your overall training time? | 1-5 Likert scale |

**Table H** – Studies 3 &4 Participant Post Intervention Questionnaire

| **Questions** | **Study 1** | **MAX** | **MAXboff** | **Study 2** | **MAXboff** | **AMRAP** |
| --- | --- | --- | --- | --- | --- | --- |
|  |  | *5 point Likert Scale score* | *5 point Likert Scale score* |  | *5 point Likert Scale score* | *5 point Likert Scale score* |
| How enjoyable was the training protocol that you were assigned to? |  | 3.3±1 | 3.5±0.9 |  | 3.7±1.2 | 3.4±1.1 |
| How easy was it to adhere to your training protocol? |  | 4.5±0.7 | 4.3±0.5 |  | 4±0.7 | 4±1.5 |
| How manageable was the workload of your training sessions? |  | 4±0.9 | 4.2±0.7 |  | 4.4±0.8 | 4.2±0.9 |
| How effective was your training protocol in covering your training needs? |  | 3.6±1 | 3.6±0.7 |  | 3.4±1.1 | 3±0.8 |
| How likely are you to use the same or a similar training protocol in the future? |  | 3±1 | 3.3±0.9 |  | 3±1 | 3.2±1.5 |
| How much did the training protocol impact current or past injuries? |  | 2.5±1 | 2.1±1.1 |  | 1.2±0.4 | 2.2±0.9 |
| How likely are you to utilise such a training approach during a deload period? |  | 2.8±1.3 | 2.5±0.9 |  | 4.2±0.8 | 3.2±1.5 |
| How likely are you to utilise such a training approach during a busy period with limited time available for training? |  | 4±0.9 | 3.6±1.3 |  | 3.6±1.3 | 4.2±1.5 |
| How much did exogenous factors (stress, lack of sleep, personal issues) affect your performance during the training intervention? |  | 3.2±1.6 | 2.8±1.4 |  | 1.4±0.8 | 2.5±1.9 |
| How confident were you at utilising the RPE scale during training sessions? |  | 4.2±0.7 | 4.2±0.7 |  | 4.4±0.5 | 3.7±0.5 |
| How likely are you to utilise the same or a similar protocol during a strength gain phase? |  | 3.1±1.3 | 3.6±0.9 |  | 2.4±1.1 | 3.5±0.5 |
| How effective was the protocol you followed in reducing your overall training time? |  | 4±1.1 | 3.8±1.2 |  | 4.6±0.5 | 4.7±0.5 |

*Results are mean ± SD

**Table I – The minimum effective dose survey responses**

| Question |  |
| --- | --- |
| **Why do you use a minimum effective dose training approach?** | **Total (% of participants)** |
| Limited time available | 47.6 |
| Reduce fatigue | 47.6 |
| Injury management | 38.1 |
| I enjoy training more with a minimum effective dose approach | 33.3 |
| Longevity in Powerlifting | 33.3 |
| I find it easier to progress using a minimum effective dose approach | 28.6 |
| Low motivation to train | 14.3 |
| I do not enjoy overreaching symptoms | 9.5 |
| Other | 19 |
| **When do you use a minimum effective training dose approach?** | **Total (% of participants)** |
| Busy periods due to exogenous factors (eg: work, studies, family) | 61.9 |
| Competition preparation | 61.9 |
| Off-Season | 42.9 |
| Deload | 33.3 |
| Other | 14.3 |
| **How long do you use a minimum effective training dose approach for (in consecutive weeks)?** | **-** |
| Mean (±SD) | 9.1±10.7 |
| Minimum | 1 |
| Maximum | 52 |
| **How often do you use a minimum effective training dose approach within a training year?** | **Total (% of participants)** |
| 1 – Not often at all | 19% |
| 2 | 19% |
| 3 | 28.6% |
| 4 | 23.8% |
| 5 – Extremely often | 9.5% |
| **In a year, how many months in total do you train using a minimum effective training dose approach?** | **Total (% of participants)** |
| 2 | 28.6 |
| 3 | 23.8 |
| 4 | 9.5 |
| 5 | 4.8 |
| 6 | 4.8 |
| 8 | 4.8 |
| 9 | 4.8 |
| 12 | 4.8 |
| Other | 14.3 |
| Mean (±SD) | 4.1±2.8 |
| **When utilizing a minimum effective dose training approach, how many days per week do you train?** | **Total (% of participants)** |
| 5 | 57.1 |
| 4 | 23.8 |
| 3 | 14.3 |
| Other | 4.8 |
| Mean (±SD) | 3.5±0.7 |
| **When utilizing a minimum effective dose training approach, how many times per week do you train the Squat?** | **Total (% of participants)** |
| 1 | 23.8 |
| 2 | 66.7 |
| 3 | 4.8 |
| Other | 4.8 |
| Mean (±SD) | 1.8±0.5 |
| **When utilizing a minimum effective dose training approach, how many times per week do you train the Bench Press?** | **Total (% of participants)** |
| 1 | 9.5 |
| 2 | 33.3 |
| 3 | 47.6 |
| 4 | 4.8 |
| Mean (±SD) | 2.5±0.7 |
| **When utilizing a minimum effective dose training approach, how many times per week do you train the Deadlift?** | **Total (% of participants)** |
| 1 | 76.2 |
| 2 | 14.3 |
| Mean (±SD) | 1.1±0.3 |
| **When utilizing a minimum effective dose training approach, how many weekly working sets do you perform for the Squat?** | **Total (% of participants)** |
| 3 | 15 |
| 4 | 20 |
| 5 | 10 |
| 6 | 35 |
| 7 | 5 |
| 8 | 15 |
| Mean (±SD) | 5.4±1.6 |
| **When utilizing a minimum effective dose training approach, how many weekly sets do you perform for the Bench Press?** | **Total (% of participants)** |
| 3 | 9.5 |
| 4 | 4.8 |
| 5 | 4.8 |
| 6 | 19 |
| 7 | 4.8 |
| 8 | 14.3 |
| 9 | 23.8 |
| 10 | 9.5 |
| Other | 9.5 |
| Mean (±SD) | 7.6±3.2 |
| **When utilizing a minimum effective dose training approach, how many weekly sets do you perform for the Deadlift?** | **Total (% of participants)** |
| 2 | 4.8 |
| 3 | 38.1 |
| 4 | 33.3 |
| 5 | 9.5 |
| 6 | 4.8 |
| 8 | 4.8 |
| Other | 4.8 |
| Mean (±SD) | 3.8±1.3 |
| **Question** | **Mean (±SD)** |
| When utilizing a minimum effective dose training approach, how many repetitions do you perform per working set for the Squat? | 3.5±1.4 |
| When utilizing a minimum effective dose training approach, how many repetitions do you perform per working set for the Bench Press? | 4.1±1.8 |
| When utilizing a minimum effective dose training approach, how many repetitions do you perform per working set for the Deadlift? | 3.6±1.3 |
| When utilizing a minimum effective dose training approach, approximately what %1RM do you use for your working sets for the Squat? | 80.5±8.5 |
| When utilizing a minimum effective dose training approach, approximately what %1RM do you use for your working sets for the Bench Press? | 80.4±6.5 |
| When utilizing a minimum effective dose training approach, approximately what %1RM do you use for your working sets for the Deadlift? | 78.3±8.8 |
| When utilizing a minimum effective dose training approach, approximately what RPE (based on RIR) are your working sets for the Squat? | 7.8±1.1 |
| When utilizing a minimum effective dose training approach, approximately what RPE (based on RIR) are your working sets for the Bench Press? | 8.1±0.9 |
| When utilizing a minimum effective dose training approach, approximately what RPE (based on RIR) are your working sets for the Deadlift? | 7.7±1.3 |
| **When training with a minimum effective dose approach, how many accessory exercises do you perform for the Squat (weekly)?** | **Total (% participants)** |
| 1 | 28.6 |
| 2 | 33.3 |
| 3 | 9.5 |
| 4 | 14.3 |
| Other | 14.3 |
| Mean (±SD) | 2.1±1 |
| **How many weekly sets do you (approximately) perform for your Squat accessory exercises?** | **Total (% participants)** |
| 1 | 5.3 |
| 3 | 36.8 |
| 4 | 10.5 |
| 6 | 15.8 |
| 8 | 5.3 |
| Other | 26.3 |
| Mean (±SD) | 4±1.8 |
| **Question** | **Mean (±SD)** |
| Approximately how many repetitions are your working sets for your Squat accessory exercises? | 9.3±2.2 |
| What RPE (based on RIR) are your Squat accessory exercises? | 7.6±0.9 |
| **When training with a minimum effective dose approach, how many accessory exercises do you perform for the Bench Press (weekly)?** | **Total (% participants)** |
| 1 | 19 |
| 2 | 23.8 |
| 3 | 19 |
| 4 | 23.8 |
| 5 | 4.8 |
| Other | 9.5 |
| Mean (±SD) | 2.6±1.2 |
| **How many weekly sets do you (approximately) perform for your Bench Press accessory exercises?** | **Total (% participants)** |
| 1 | 4.8 |
| 3 | 23.8 |
| 4 | 9.5 |
| 6 | 14.3 |
| 7 | 4.8 |
| 8 | 4.8 |
| 10 | 4.8 |
| Other | 33.3 |
| Mean (±SD) | 7.3±6.6 |
| **Questions** | **Mean (±SD)** |
| Approximately how many repetitions are your working sets for your Bench Press accessory exercises? | 9.2±2.9 |
| What RPE (based on RIR) are your Bench Press accessory exercises? | 7.7±0.7 |
| **When training with a minimum effective dose approach, how many accessory exercises do you perform for the Deadlift (weekly)?** | **Total (% participants)** |
| 1 | 47.6 |
| 2 | 14.3 |
| 3 | 9.5 |
| 5 | 4.8 |
| Other | 23.8 |
| Mean (±SD) | 1.6±1.1 |
| **How many weekly sets do you (approximately) perform for your Deadlift accessory exercises?** | **Total (% participants)** |
| 1 | 5 |
| 2 | 5 |
| 3 | 40 |
| 4 | 5 |
| 5 | 10 |
| 6 | 10 |
| Other | 25 |
| Mean (±SD) | 4.2±3.1 |
| **Question** | **Mean (±SD)** |
| Approximately how many repetitions are your working sets for your Deadlift accessory exercises? | 8.8±2 |
| What RPE (based on RIR) are your Deadlift accessory exercises? | 7±1.3 |
| **Question** | **Mean (±SD)** |
| How much did your Squat 1RM strength change after training with a minimum effective dose training approach (in kg)? | 14.8±11 |
| How meaningful do you consider the change in your Squat 1RM Strength? | 3.3±1 |
| How much did your Bench Press 1RM strength change after training with a minimum effective dose training approach (in kg)? | 7.7±8.3 |
| How much did your Bench Press 1RM strength change after training with a minimum effective dose training approach (in kg)? | 2.9±1.3 |
| How much did your Deadlift 1RM strength change after training with a minimum effective dose training approach (in kg)? | 14.2±15.9 |
| How meaningful do you consider the change in your Deadlift 1RM Strength? | 3±1.5 |

**Figures**

**Studies 3&4

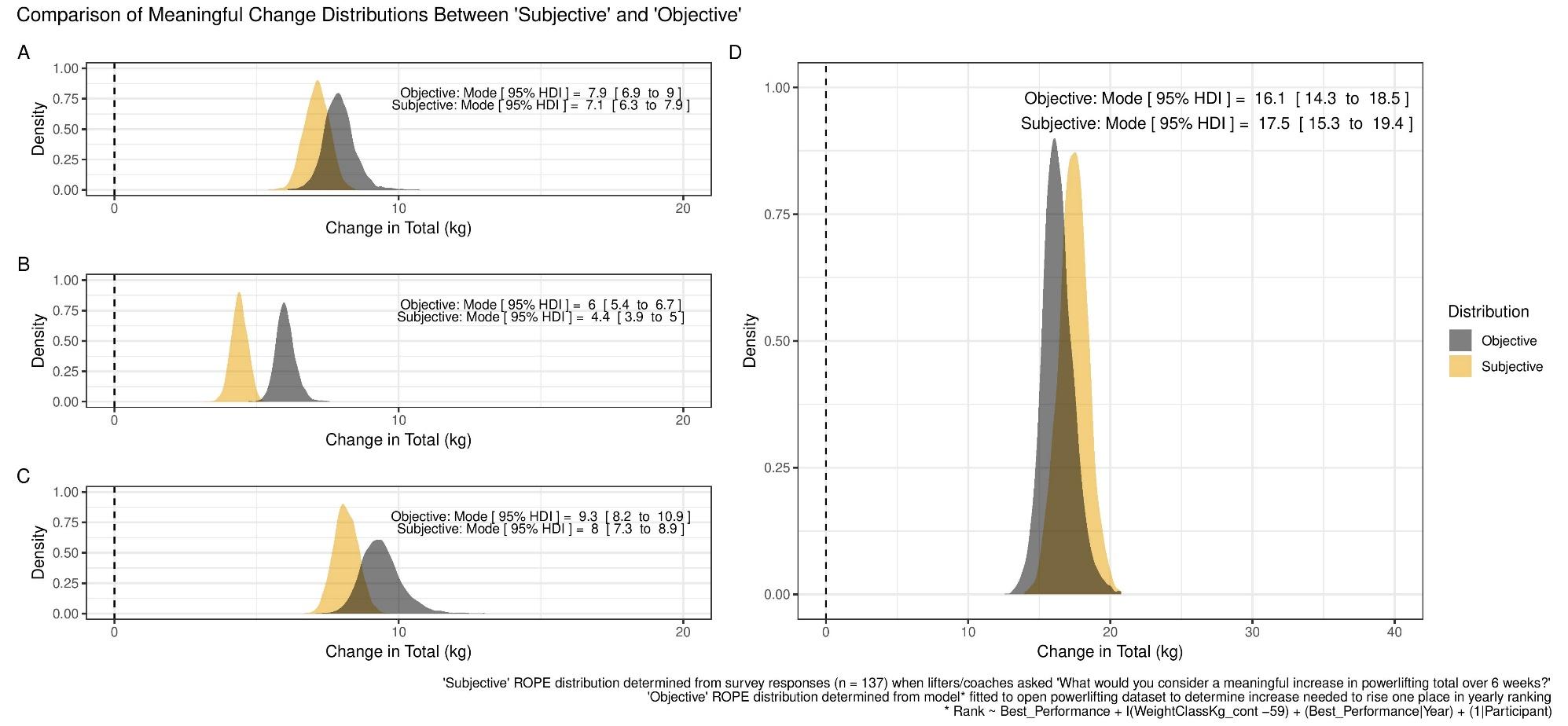
**

**Figure A -** ‘Objective’ ROPE which was determined from modelling of the open powerlifting dataset (https://www.openpowerlifting.org/) to determine the increase in SQ, BP, DL, and PL total bests to result in an increase of one position in yearly rankings within weight classes.

**Thematic analysis Study 1 – Themes & Subthemes with below 50% sample representativeness**

**Broad Theme:** The concept of a minimum effective dose for 1RM strength

**Subtheme:** Important & interesting concept

PL athletes

30% of PL athletes mentioned that the concept of METD is an important & interesting concept. They expressed that they “*think the minimum effective dose is a particularly important concept when it comes to powerlifting”* and that “*it’s a great concept and I have been kind of following that approach on and off for a good time in my lifting years”.* Some also mentioned that they had not appreciated the concept of a minimum effective dose before and how important it could potentially be as they could *“definitely appreciate that idea and that concept because like, I didn't really get how important it could be”*.

PL Coaches

27% of PL coaches expressed that the concept of METD is an important and interesting concept. Similarly to the PL athletes they expanded on how they *“think conceptually, it's great. We should be looking at this. We should be researching and trying to find out more information about it”* and that they *“think that it's something that we, as coach practitioners should, should strive for”*. Some coaches also expanded on how a METD is *“it's useful more as a theory rather than a number. Because there are so many things that affect recoverability and continuality, coming up with an actual number that is reliable and you can return to probably does not have as much utility in the same way as saying 123, like we said before, but considering it as a concept, and having kind of a range to work from minimum on one side and maximum on the side, it makes perfect sense. And so, in that light, I can easily understand where we might imagine a minimum effective dose and maximum”*.

**Subtheme:** Important concept to ensure long-term success

PL athletes

Only 10% of PL athletes, so 1 PL athlete, expressed that the concept of a minimum effective training dose may be important to ensure long-term success in PL. Despite being just one out of the 10 PL athletes interviewed, this athlete is a current ATWR holder for the DL and PL total and ranks in the top 10 RAW PL athletes worldwide, regardless of weight class, competition year, federation or sex. They expressed that “*finding what your minimum effective dose is, is, you know, one of the one of the most important variables to in order to have a long-lasting career, [as there is] so much beating that your body can take. I think the minimum effective dose highly varies from individual to individual, and most importantly, has to do with again, the level of experience of a lifter. As I've evolved as an athlete, I've noticed that I can get away with way less work and way less training sessions to both maintain and increase my one rep max”.*

PL Coaches

Contrastingly to the PL athletes, 27% of PL coaches expressed that the concept of a minimum effective training dose may be important to ensure long-term success in PL. The expanded on how using a minimum effective training dose approach may reduce help with sustainability by avoiding overuse injuries as *“there are periods where you're trying to do the minimum effective dose. I like, again, it might bore you a bit, but I had a conversation with a lifter the other day, a female lifter. And, you know, we found that getting her to bench for up to five, but generally four times a week, we get great results, but had to try and explain to her because I brought it back down to three times. And she wants four or five, you know, she wants to get stronger. But at the same time, I said, look, the truth is, it's not sustainable long term. We're not near a competition. Let's bring it back. Let's desensitize you’re a little and you're still getting gains [albeit suboptimal] from just the minimum effective dose of training”.* Understanding when it makes sense to decrease training workload, but still make progress is important as *“the big reality of things, at least from what I've gathered over my years is that you you might be able to push and again, we see this in the literature depending on what you've read, maybe by you know, pushing training volume a little bit harder. You may be able to get a little bit more progress. But the thing is, that the more you put somebody under the bar, the more of a chance there is of injury, whether that's, you know, acute or chronic”*. The potential effect of METDfor long-term success from a psychological perspective was also mentioned as *“component of it the psychological aspects where, you know, you don't want people to burn out mentally, either you want because I think the more you do it, and the more people you train, the more you realize that people need to kind of be hungry to train, I think that gets kind of bled out of them quite quickly if they're a little bit older, and if the training frequency is too high. So in terms of the minimum effective training dose, yeah, I think having a little bit more space potentially doing a little less work, volume wise per session is preferable”*.

**Broad Theme:** The minimum effective dose for 1RM strength – in practice

**Subtheme:** The BP requires a higher training frequency/volume than the SQ&DL

PL athletes

30% of PL athletes expressed that the BP requires a higher training frequency than the SQ&DL in the context of METD. PL athletes made comments on how they have *“personally seen I need to train it very frequently and with high intensities, at least one set close to rpe8 or 9 or 90%1RM, 3-4 times per week”* and how they would make different training adjustments to the BP in comparison to the SQ&DL explaining that *“and for bench, that's my weakest lift, I think I would need at least two bench sessions just to maintain”*. An athlete followed his comment about the BP needing a higher training frequency by explaining that he thinks he has *“been conditioned to feel that bench needs a higher [training] frequency”*.

PL Coaches

22% of PL coaches expressed that the BP requires a higher training frequency than the SQ&DL in the context of METD. Some PL coaches explained how they felt that *“for benchpress I think [PL athletes] may need to train that a little bit more even”* and that *“the bench press on the other side, I place it on top [compared to SQ&DL], meaning it’d need the most amount of sets for one to make gains. When a lifter is doing less sets than what’s needed throughout the week, I could somehow say that the bench tends to look detrained, like they’d somehow forget the motor patterns”*. The idea that the BP requires more training volume than the SQ&DL was also expressed when attempting to quantify the number of weekly sets that each powerlift would need to progress “*what I probably see is like the least amount of work we have to do see progress in each lift will be sort of around the like 10 set mark on maybe on bench and like less than 10 sets on squats and deadlifts”*.

**Subtheme:** RPE 8+ for the main sets

PL athletes

Despite a few PL athletes mentioning that they would implement heavy load sets when training with a minimum effective dose, only 10% of PL athletes (1 participant) referred to the RPE of the main working sets. They mentioned that the *“hard set”* would be *“RPE 9, plus or minus 1”*.

PL Coaches

27% of PL coaches expressed that the RPE of the main sets would be 8+. The PL coaches mentioned that *“if I'm designing a block for a lifter, and the only thing that I'm worried about is increasing their 1RM strength in those three movements, I would want the reps to be only singles, if not like, maybe doubles. And they would all be at a very high intensity. So probably like an RPE of around 8 to 9 on all those sets”* and also drew on past experiences where they had *“done exactly what you're talking about, I have typically done two to four singles per week on the squat and bench press and I've reliably seen an increase and so it's just literally whatever volume requires [the athlete] to work up to that single at somewhere between 8 to 10 RPE and then one to two singles on a deadlift and most cases will get the job done”*.

**Subtheme:** SQ trained once per week

PL Athletes

30% of PL athletes expressed that they believed training the SQ once per week would be sufficient in the context of METD. PL athletes expressed that they *“think that I could probably do one heavy session of squatting a week”* and that *“it would be one squat session a week, where I work up to either a top set, you know, a low rep set to one, two or three reps”*.

PL Coaches

Only 11% of PL coaches made specific comments about the training frequency of the SQ being once per week with statements like *“you can certainly do, you know, three sessions a week one squat one bench one deadlift and make progress”* and *“it can definitely be as frequent as training each lift once per week”*.

**Subtheme:** SQ trained twice per week

PL Athletes

The subtheme of squatting twice per week did not appear in any of the PL athletes’ interviews.

PL Coaches

22% of coaches expressed that they believed training the SQ twice a week would be sufficient in the context of METD. They made statements like *“with twice per week squatting, twice per week benchpress and one time per week deadlifting, I think I could still get the job done”* and also expressed that training frequency will *“probably be sort of like three days of training potentially and for most people will probably be in terms of frequency, like maybe two days of squats, minimum of like two days of bench and one day of deadlifting”*.

**Subtheme:** BP trained once per week

PL Athletes

The subtheme of BP trained twice per week did not appear in any of the PL athletes’ interviews.

PL Coaches

Only 11% of PL coaches mentioned training the BP once per week as being sufficient in the context of METD for 1RM strength. They mentioned that they believed METD can be *“very low to see some improvement in each of those three lifts and it can definitely be as frequent as training each lift once per week”* and similarly to squatting once per week they made statements like *“you can certainly do, you know, three sessions a week one squat one bench one deadlift and make progress”*.

**Subtheme:** BP trained 2-3 times per week

PL athletes

Only 10% of PL athletes made comments on the training frequency of the BP in the context of METD. The 1 PL athlete expressed how they felt that their BP would require at least a 2 times per week training frequency to simply maintain strength. This quote was also presented under the subtheme of *“the BP needs a higher training frequency than the SQ&DL” where the PL athlete said “and for bench, that's my weakest lift, I think I would need at least two bench sessions just to maintain”*.

PL Coaches

22% of PL coaches expressed that the BP could benefit more from higher training frequencies while mentioning that a training frequency of 2-3 times per week may be a sufficient the BP. As shown above they said that *“with twice per week squatting, twice per week benchpress and one time per week deadlifting, I think I could still get the job done”* and that *“the bench press on the other side, I place it on top [compared to SQ&DL], meaning it’d need the most amount of sets for one to make gains. When a lifter is doing less sets than what’s needed throughout the week, I could somehow say that the bench tends to look detrained, like they’d somehow forget the motor patterns”.*

**Subtheme:** DL trained 1 time per week

PL Athletes

20% of PL athletes expressed that the DL only needs one training session per week in the context of METD. The expressed that in the past they have *“made progress squatting and dead lifting only once per week”*.

PL Coaches

27% of PL coaches expressed that the DL only needs one training session per week in the context of METD. They made statements like *“I think [METD] is very low to see some improvement in each of those three lifts can definitely be as frequent as training each level. once per week”*.

**Broad Theme:** The minimum effective dose for 1RM strength – length

**Subtheme:** As long as it remains effective

PL Athletes

The subtheme of “as long as it remains effective” did not appear in any of the PL athletes’ interviews.

PL Coaches

22% of coaches expressed that they would implement a minimum training dose approach for as long as it remained effective for. They made statements like *“let's see what the minimum it is that you have to do and let's just try to ride that out as long as we can. And maybe they hit a wall in six weeks, maybe, you know, maybe the gains keep coming for six months”* and that their instinct regarding the length of effectiveness *“would be indefinitely really. You know, it depends on a lot of other factors. But, I don't think necessarily you just be able to last six weeks and go, okay, right, we can't do this protocol anymore. I mean, I think we could do it for a fairly long time without having to change too much when obviously you need to be rotating various things in and out and the rest of their training from accessory weightlifting and everything else, but, you know, if you're programming intelligently, there’s no reason why you couldn't maintain that and avoiding injury then yeah. And if anything, it might be preferable”*

**Broad Theme:** When to use the METD

**Subtheme:** When time is limited

20% of PL athletes expressed that the concept of a minimum effective training dose may be particularly useful for individuals with limited time available. They mentioned that “For example if you were prepping for a meet but your daily schedule wouldn’t allow you to do as much training as you’d want to. I believe this is where you should seek your minimum effective training dose” and that “it may be something worth implementing when athletes get to the point in their career where they can't train for five to eight hours a week and they need to make a change and this information, regarding METD training, is going to be amazing”.

PL Coaches

27% of PL coaches expressed that the concept of a minimum effective training dose may be particularly useful for individuals with limited time available. They mentioned that they would potentially not implement such a training approach *“for someone who's, you know, whose life is in order, they can recover well, and they're just like, super enthusiastic about powerlifting, a minimum effective dose approach really isn't much of a consideration but for someone for whom powerlifting is a bit of a lower priority or they just have a lot of stuff going on in their life that impacts how much they can get in gym or how much they can recover from, that’s where [a METD approach could potentially be appropriate]”.* They also mentioned that a minimum effective training dose approach would be appropriate for *“periods where the athlete is busy or moving, or maybe they started grad school. So periods where the athlete either doesn't have a lot of time, or has high stress outside of the gym, or something like that would be a period where I'd be comfortable using this”* and *“if somebody only has one hour to train three days per week, then something like minimum effective dose becomes a good conversation topic”*.

**Subtheme:** Pre-Competition

PL Athletes

30% of PL athletes expressed that they may incorporate METD training pre-competition. A PL athlete mentioned that if someone was *“peaking for a meet and you aimed to make any, even suboptimal, strength gains then yes I’d say it has some value in implementing it”* while another PL athlete drew on their success with METD training pre-competition saying that they *“just switch to doing less and that has even worked for competition preparation for me”*.

PL Coaches

Only 1 coach (5%) expressed that they would incorporate METD training pre-competition. They made the following statement “*Yeah, pre comp. That's normally when I like to play with it. So I normally do that in two time periods. If someone is a little beat up coming from a volume block, like if they are more jacked up from the volume phase, going into the peaking before the comp, and I really think we need to pull back, but I also want to still, you know, get them specific training prior prior to the comp. I've done this, to get them an overall kind of physical recovery but still exposure to the specific skills.”*.

**Subtheme:** When not feeling 100%

PL Athletes

20% of PL athletes said that they would use a minimum effective training dose approach when injured, to better manage injuries while still make some progress. They drew on past experiences experiment with METD and made statements like *“when I was prepping for the 2018 meet, because I was having patellar tendon pain, I did one top set of one or two reps in the squat per week and I ended up tying my then all best, which I’ve hit months ago when I was training under normal circumstances. So, I’d say I did follow that approach, I had no increases but I tied my all time best while being detrained and hitting one top set of one or two reps per week”*. Other PL athletes said that they realized that *“increasing workload after a competition I'm realized that it isn't really, that's not how it works, you know, you can't really increase everything fast. So minimizing it sounds fun, [I would use a minimum effective dose approach] when I just don't feel like as well, when I'm burnt up, by mostly after competitions”.*

PL Coaches

Only 11% of PL coaches expressed that they would use a minimum effective training dose approach when not feeling 100%. 1 PL coach expressed that *“outside the context of injured athletes [METD] is not something that I am ever aiming for”*. The other PL coach expanded on how using such an approach could be beneficial for a PL athlete returning from injury stating that *“another time that's been very successful is when I've had an athlete come back from an injury. So they're ready to start squatting and deadlifting. Again, and I know and I basically want to give them the minimal amount to get back to where they were. And then they often plateau within 10% of their old numbers just on a couple singles a week. And I think that's a really useful tool to give them that graded exposure. And, and then you can, you know, put that energy into something else”*.

**Broad Theme:** The minimum effective dose for 1RM strength – additional considerations

**Subtheme:** Suboptimal – “why do less when you can do more”

PL Athletes

20% of PL athletes expressed that training with a minimum effective dose approach would not always be preferable as they could not justify *“doing less when they could be doing more”.* One PL athlete, who is a current ATWR record holder on the BP and multiple IPF world champion, said that they would *“do everything it takes to get better”*. Another PL athlete, who is a multiple IPF National champion, mentioned that *“when competing, [I’ll do] whatever my body can handle” and that “I don't know any of us, that wouldn't just work harder because we're competitors”*.

PL Coaches

**Subtheme:** Change of mindset from more training volume is always better

PL Athletes

30% of PL athletes expressed that over time, their mindset changed from thinking that more training volume is always better. One PL athlete stated *“I was pushing a lot more volume on myself in general, like I was trying to do a lot more in the gym, than maybe I should have been doing”* and that they had *“heard of the concept, and I don't think I really fully appreciated it”.* Similarly, another PL athlete explained how *“As I've evolved as an athlete, I've noticed that I can get away with way less work and way less training sessions to both maintain and increase my one rep max”*.

PL Coaches

The subtheme of *“change of mindset from more training volume is always better”* did not appear in any of the PL athletes’ interviews.

**Thematic analysis Study 2 – Themes & Subthemes with below 50% sample representativeness**

**Broad Theme:** Meaningfulness of strength changes in 6 weeks

**Subtheme:** A 5-10 kg increase for the SQ may be meaningful

PL Athletes

30% of PL athletes expressed that a 5 to 10kg increase on the SQ may be meaningful. One PL athlete mentioned that they *“would probably just expect five to 10 pounds in the benchpress and my squat, probably 15 pounds and my deadlift probably 15”* and continued to say “*that would be in a full cycle. I usually I do 10-12 weeks. So I guess in a six week I would half that”*. Others simply provided a quantifiable answer regarding the strength change that they would consider meaningful for the SQ, without expanding further stating that “for the squat would be, I guess, five to 10 [kg]”. A PL athlete further elaborated on the above subtheme of “any change is meaningful” by saying that *“I would say that for me personally any change would be meaningful in just 6 weeks. But if I had to put a number on it I would say that around 10 kilos for the squat”*.

PL Coaches

33% of PL coaches expressed that an approximate increase of 5 to 10kg increase on the SQ may be meaningful. They expressed that *“it would be relatively meaningful to get a five to 10 kilo gain, you know, on maybe this on the squat and the deadlift”*. A PL coach expanded on their answer and explained how *“in a six week period, I would say something like a five kilos increase in squat, probably two and a half in bench and five to seven and a half in deadlift would be fairly [standard]. I would say something along those lines is fairly standard, sometimes a lot more sometimes none. You know, there's a bell curve, well, maybe not quite a bell, but there's obviously a distribution of how much progress people make. So yeah, I'd say probably approximately, you know, somewhere between two and a half to seven and a half on squat”*. Others simply stated that *“being able to add five to seven and a half kilos per six weeks I think is a is a very meaningful change [and is] something that you would like be able to look back over that block and say this went really, really well”*.

**Subtheme:** A 2.5-5kg increase on the BP may be meaningful

PL Athletes

20% of PL athletes expressed that a 2.5-5kg increase on BP may be meaningful. They stated that *“maybe around 5 or so for the bench press”* would be meaningful and that *“[for the] bench five [kg would be meaningful]”*.

PL Coaches

33% of PL coaches expressed that a 2.5-5kg increase on BP may be meaningful. They stated that *“it would be relatively meaningful to get a five to 10 kilo gain, you know, on maybe this on the squat and the deadlift and maybe a kind of 2.5 to five on the bench”* and that *“in a six week period, I would say something like a five kilos increase in squat, probably two and a half in bench”*. Other PL coaches expressed how they would be satisfied with less of an increase on the BP compared to the SQ&DL, stating that *“if we said something like, seven and a half kilos for the squat and the deadlift maybe, and then a bit less for the bench”*.

**Subtheme:** A 5-10kg increase on the DL may be meaningful

PL Athletes

30% of PL athletes expressed that a 5-10kg increase on the DL may be meaningful. They stated that *“a 5kg increase in the deadlift and 10kg to be very meaningful”* and that *“if I had to put a number on it I would I would say that around 10 kilos for the squat and the deadlift”.*

PL Coaches

Only 16% of PL coaches expressed that a 5-10kg increase on the DL may be meaningful, stating *“if we said something like, seven and a half kilos for the squat and the deadlift”* and *“It would be relatively meaningful to get a five to 10 kilo gain, you know, on maybe this on the squat and the deadlift”*.

**Subtheme:** A 2.5-5kg increase per lift may be meaningful

PL Athletes

Only 1 PL athlete expressed that a 2.5-5kg per powerlift would be regarded as meaningful, stating that “2.5-kg per lift” when asked about meaningful strength changes over 6 weeks.

PL Coaches

One PL coach expanded on how the athlete’s level may make a 2.5-5kg increase per powerlift meaningful, saying *“I would say that it depends on the experience with the athlete. For very advanced athletes, like if we're talking about Brett Gibbs, as an elite athlete, I would say being able to use soreness, something as small as you know, one to 2.5 kilos on any one of those lifts is a meaningful over a six week period and effect It's probably more more than I might might typically expect”.* Similarly to the one PL athlete, a PL coach simply stated *“I would say two and a half to five kilos per lift”.*

**Subtheme:** A 2+% increase per powerlift may be meaningful

PL Athletes

The subtheme of *“A 2-3% increase per powerlift may be meaningful”* did not appear in any of the PL athletes’ interviews.

PL Coaches

33% of PL coaches expressed that a 2-3% increase per powerlift may be meaningful. They expanded on how for non-novices, a 2-3% increase per powerlift will be meaningful, stating that “*if someone's already, like a national or international level lifter, if they're getting 2% 3% stronger in six weeks, I'm super stoked about that”* and that *“I think I would probably say something like, you know, one to 2% would be would be pretty good for non novice power lifters over a six week period would be pretty solid per lift”*. Some other PL coaches mentioned that for any PL athlete they *“believe that a 2.5-7.5% increase in strength would be very good, over 6 weeks”*.

**Broad Theme:** Factors affecting the magnitude of meaningfulness of strength changes in 6 weeks

**Subtheme:** Time of the training season (eg: competition vs off-season)

PL Athletes

40% of PL athletes expressed that meaningfulness of strength changes in 6 weeks will depend on the time of the training season. They stated that “in the offseason, we usually make a lot more progress. So meaningful would be larger in the offseason. And during competition season meaningful would be basically maintaining or anything above maintenance” and that *“[a strength change] is more meaningful, like when you're finishing competition, because it's, well, it counts for more”.* Other PL athletes expanded further and explained that there are other times during one’s training season where meaningfulness can change, stating that *“had I already been detrained for any reason (for example: quarantine), or if I was coming from a less specific training block, if I had been regaining strength I already had, or if I was following a hypocaloric diet or coming back from injury, so yeah if I was already at my all time best and I added on top of them, I’d be excited about it. If I, for any reason, had any performance drop, or if I was gaining weight, I’d still be happy about it but I wouldn’t consider them as important”*.

PL Coaches

The subtheme of *“Time of the training season (eg: competition vs off-season)”* did not appear in any of the PL coaches’ interviews.

**Subtheme:** Bodyweight of the athlete

PL Athletes

The subtheme of *“Bodyweight of the athlete”* did not appear in any of the PL coaches’ interviews.

PL Coaches

33% of PL coaches expressed that the bodyweight of the athlete may impact the magnitude of meaningfulness in terms of strength changes in 6 weeks. They expressed that *“I think a big one you're going to deal with right off the bat is going to absolutely be bodyweight. Because you're talking about magnitude of increase on someone who's got a 600 pound squat, a five pound increase on them is not that significant. Whereas a five pound increase on someone who's got a 250 pound squat is pretty damn significant. Like a, like a female lifter”* and *“It might depend on body weight. So lighter, lighter athletes, as a result may gain less on their overall total training experience. So more experienced athletes may expect to gain less per unit of time. Training experience to so newer athletes versus more experienced athletes as well”*. Some PL coaches further expanded on the point of bodyweight affecting the meaningfulness and magnitude of strength change is 6 weeks by also referring to the biological sex of the athletes. One PL coach stated that *“I do find that men tend to see bigger change. It might not be a male female thing, it might just be a percentage of 1RM. thing you know that, uh, you know, a smaller absolute change will be a higher percentage of 1RM for a smaller person. Okay. Yeah. So, size of the person may matter, experience level will”.*
